# Prognostic Signature Development on the Basis of Macrophage Phagocytosis-Mediated Oxidative Phosphorylation in Bladder Cancer

**DOI:** 10.1155/2022/4754935

**Published:** 2022-09-29

**Authors:** Genyi Qu, Yong Xu, Zhenquan Lu, Haibo Nie, Cheng Tang, Jian Hou, Xiangyang Wen

**Affiliations:** ^1^Department of Urology, Zhuzhou Central Hospital, Zhuzhou 412007, China; ^2^Division of Urology, Department of Surgery, The University of Hongkong-Shenzhen Hosipital, Shenzhen, 518000, China

## Abstract

**Background:**

Macrophages are correlated with the occurrence and progression of bladder cancer (BCa). However, few research has focused on the predictive relevance of macrophage phagocytosis-mediated oxidative phosphorylation (MPOP) with BCa overall survival. Herein, we aimed to propose the targeted macrophage control based on MPOP as a treatment method for BCa immunotherapy.

**Methods:**

The mRNA expression data sets and clinical data of bladder cancer originated from Gene Expression Omnibus (GEO) and The Cancer Genome Atlas (TCGA) data set. A systematic study of several GEO data sets found differentially expressed macrophage phagocytosis regulators (DE-MPR) between BCa and normal tissues. To discover overall survival-associated DE-MPR and develop prognostic gene signature with performance validated based on receiver operating curves and Kaplan-Meier curves, researchers used univariate and Lasso Cox regression analysis (ROC). External validation was done with GSE13057 and GSE69795. To clarify its molecular mechanism and immune relevance, GO/KEGG enrichment analysis and tumor immune analysis were used. To find independent bladder cancer prognostic variables, researchers employed multivariate Cox regression analysis. Finally, using TCGA data set, a predictive nomogram was built.

**Results:**

In BCa, a four-gene signature of oxidative phosphorylation composed of PTPN6, IKZF3, HDLBP, and EMC1 was found to predict overall survival. With the MPOP feature, the ROC curve showed that TCGA data set and the external validation data set performed better in predicting overall survival than the traditional AJCC stage. The four-gene signature can identify cancers from normal tissue and separate patients into the high-risk and low-risk groups with different overall survival rates. The four MPOP-gene signature was an independent predictive factor for BCa. In predicting overall survival, a nomogram integrating genetic and clinical prognostic variables outperformed AJCC staging. Multiple oncological features and invasion-associated pathways were identified in the high-risk group, which were also correlated with significantly lower levels of immune cell infiltration.

**Conclusion:**

This paper found the MPOP-feature gene and developed a predictive nomogram capable of accurately predicting bladder cancer overall survival. The above discoveries can contribute to the development of personalized treatments and medical decisions.

## 1. Introduction

Bladder cancer (BCa) has been recognized as one of the most prevalent cancers of the genitourinary system, as well as one of the top 10 cancers worldwide [1]. BCa is divided into muscle-invasive bladder cancer (MIBC) and non-muscle-invasive bladder cancer (NMIBC) in accordance with whether it invades the bladder's musculature [2]. Surgery and postoperative Bacille Calmette-Guérin (BCG) infusion and other immunotherapies have been adopted to treat BCA, whereas there is still ~20% of BCa cases with their bladder muscle invaded. MIBC exhibits a high rate of recurrence, progression, and mortality even based on the existing treatments [3, 4]. Patients with urothelial cancer of any stage had a five-year overall survival rate of 66-68 percent [5]. The optimal preclinical model and the absence of precise biomarkers for early cancer identification hinder effective clinical therapy of BCa. As a result, it is critical to look at various types of cell death to overcome tumor cell resistance and find novel and effective biomarkers for BCa early diagnosis. The molecular mechanism of the incidence and malignant progression of bladder cancer should be understood in depth to develop more effective treatment methods, so as to facilitate the clinical prognosis of patients. The cancer niche is heavily dependent on inflammatory cells [6]. Varying numbers of macrophages, the major component of leukocyte infiltration, exist in all tumors [7]. Macrophages have a vital role in tumor inflammation. TAM supports tumor growth on multiple levels (e.g., increasing genomic instability, cultivating cancer stem cells, paving the route for metastasis, and taming protective immunity). TAMs express checkpoint triggers, thus controlling T-cell activation and becoming the target of checkpoint-blocking immunotherapies. Macrophage-centered therapies consist of techniques that trigger extracellular death or phagocytosis of cancer cells (e.g., tumor recruiting and survival prevention, functional re-education anticancer, M1-like mode, and tumor-targeting monoclonal antibody) [8]. Phagocytosis is of great significance in neutralizing and terminating infections, whereas it is also beneficial to establish, balance, and manifest noninfectious illnesses. The process consists of the clearance of apoptotic cells, turnover of senescent erythrocytes, monitoring of tumors, elimination of cell fragments, and synaptic pruning after injury [9–15]. Imbalance between professional and nonprofessional phagocytes can result in the accumulation of autoimmune, developmental defects, and toxic proteins [16, 17]. We have previously found that the polarization of macrophage can weaken attenuating inflammatory scar stenosis in New Zealand rabbits [18]. Our recent study has also found the effect exerted by M2-TAMs on the promotion of bone metastasis, chemotherapy, and endocrine therapy for resistance in prostate cancer (PCA), and the immunotherapy in patients with PCA can be affected by the regulation of macrophage polarization [19].

In macrophages, NO is synthesized by iNOS, while superoxide is mainly produced by NADPH oxidase. The reaction of superoxide with NO leads to the formation of peroxynitrite in vivo. This iNOS-derived peroxynitrite results in nitrotyrosine formation, increased antibacterial activity, and cytotoxic actions of macrophages [20]. Recent evidence indicates that peroxynitrite contributes most of the cytotoxicity of resident macrophages. Peroxynitrite interacts with lipids, DNA, and proteins via direct oxidative reactions or indirect radical-mediated mechanisms. These reactions trigger cellular responses ranging from subtle modulations of cell signaling to overwhelming oxidative injury. In vivo, peroxynitrite generation has been attributed to inflammatory diseases such as stroke, myocardial infarction, chronic heart failure, diabetes, circulatory shock, cancer, and neurodegenerative disorders [21]. Identification and identification of phagocytic regulatory factors are of great significance in the analysis of tumor cell phagocytic mechanism.

In this paper, the aim is to fully describe the dysfunction and regulatory role of phagocytosis regulators in tumor and to analyze the regulatory pattern in tumor. To find DEGs, we combined two bladder cancer data sets from the GEO database. Overall survival-associated DE-MPOP were identified using single variable and Lasso-Cox regression analysis, and predictive gene features were proposed using TCGA BLCA data set and clinical data. The prognostic genetic characteristics were validated using external data sets. The molecular mechanism of gene identity, the correlation of tumor immunity, and its potential in conducting immunotherapy were also studied. Multivariate Cox survival analysis was adopted to identify independent predictive markers for overall survival. Overall survival was predicted using a prognostic nomogram that combined prognostic gene signatures and clinical prognostic variables. Overall, our prognostic gene signature and nomogram are able to reliably predict BCa overall survival.

## 2. Materials and Methods

### 2.1. Gene Expression and Clinical Data Collection

Search and download bladder cancer mRNA expression and clinical data from GEO (https://www.ncbi.nlm.nih.gov/GEO/) using the keywords “bladder cancer,” “BLCA,” and “Bca.” The following stage of screening compromised “Homo sapiens” and “Expression analysis by array.” The search also eliminated “cell lines” and “xenografts.” For DEG analysis, the gene expression microarray data sets GSE13507 and GSE69795 were chosen and downloaded. The above data sets satisfy the following requirements: (1) human BCa tissue samples, (2) tumor and nontumor bladder control tissue samples, and (3) a total of 30 samples. The associated follow-up information of GSE13507 and GSE69795 with 246 and 61 BCa tissues, respectively, was downloaded for subsequent prognostic gene signature verification [22, 23]. Using the annotation files given by the manufacturer, match probes to gene symbols. If multiple probes match a gene, the median rank values account for the expression values. Data normalized by Robust multiarray average (RMA) are logarithmically converted for further analysis. The Cancer Genome Atlas (TCGA) data sets (https://portal.gdc.Cancer.gov/; 2019.05.20) were adopted to get clinical information on normalized RNA sequencing data as Transcript per million (TPM) and BLCA samples, which consist of 418 tumor samples and 38 normal tissue samples (Supplementary Table 1). For subsequent analysis, the normalized gene expression data from TCGA BLCA data set were transformed logarithmically.

### 2.2. Identification of Phagocytosis Regulatory Factor Data Sets

Phagocytosis regulatory factor data sets have been collected due to the great significance to identify and characterize phagocytosis regulators in tumors [24, 25]. 271 genes were obtained by crossing with TCGA-BLCA gene set for further analysis. Using the ssGSEA algorithm to obtain the macrophage enrichment score in samples and the Pearson correlation score as well as the expression of phagocytosis regulatory factor.

### 2.3. Functional Analysis of Phagocytosis Regulators

DEG's putative biological processes, cellular components, and molecular activities were investigated using GO enrichment and KEGG pathway analysis. The signal pathways were strongly correlated with what David (https://DAVID.ncifcrf.gov/) found [26]. This research makes use of annotation, visualization, and a large discovery database (David did a functional enrichment analysis on MPOP variables). Furthermore, using the R package “cluster Profiler” and data from the Kyoto Encyclopedia of Genes and Genomes (KEGG), functional analysis of biological processes (BP), molecular functions (MF), and cellular components (CC) regulated by macrophage phagocytosis regulators was done. The cut-off for *P* values was established at *P* < 0.05.

### 2.4. Differential Phagocytosis Regulator Expression in Tumors

To investigate the differential expression of phagocytosis regulators in malignancies, we evaluated the expression levels of 233 cellular phagocytosis regulators in tumor tissue and normal tissue. The “limma” package of R language version 4.1.1 was adopted to find differentially expressed phagocytosis regulators between BLCA and normal bladder specimens, and the screening requirements were |log fold‐change| > 1.0 and the false discovery rate (FDR) < 0.05. The expression matrices of differentially expressed lncRNAs were visualized by the heatmap software package This paper then performed PCA analysis based on the R packages “FactoMineR” and “factoextra” to explore the discriminatory power of phagocytosis regulators in tumors.

### 2.5. Identification of Prognostic-Associated Phagocytosis Regulators and Construction of Prognostic Signature

MPOP correlated with overall survival were found using TCGA-BLCA data set. A complete examination of the GEO data set was utilized to evaluate the expression levels of macrophage phagocytosis regulators using univariate Cox regression analysis. MPOP with a statistical significance of *P* < 0.05 were included in further analyses. Lasso-Cox regression analysis was conducted using 10-fold cross-validation based on the “glmnet” package in R to further minimize the number of MPOP with the optimal predictive performance in the selected panel. The regression coefficients from the Lasso-Cox regression model multiplied by the mRNA expression levels of patients with bladder cancer were adopted to create a predictive genetic profile. The specific model equation was as follows:

(1) $$\begin{array}{c} \text{Risk score}=-0.20*\exp\left(\text{PTPN}6\right)-0.02*\exp\left(\text{IKZF}3\right)+0.09*\exp\left(\text{HDLBP}\right)+0.01*\exp\left(\text{EMC}1\right). \end{array}$$

Patients were assigned into the low-risk and low-risk groups in accordance with the optimal cut-off time for prognostic gene signatures [27]. The performance of the prognostic genetic signature was evaluated using Kaplan-Meier analysis, area under the receiver operating characteristic (ROC) curve (AUC), and calibration plots comparing predicted and observed overall survival. As a control, the relationship between AJCC (e.g., stage, grade, T-stage, sex, age, and smoking) expression and clinical characteristics was analyzed. The predictive gene signature's performance was compared to three previously identified gene signatures [28–30]. For external validation, the GSE13507 and GSE69795 data sets with comprehensive clinical information were employed. The prognostic gene signatures were adopted to compute risk ratings. The risk score's ability to predict overall survival was confirmed using AJCC staging as a control.

### 2.6. Predictive Nomogram Construction and Validation

After examining the association between all independent prognostic indicators and relevant clinical parameters, a stepwise Cox regression model was adopted to predict 1-, 2-, and 3-year overall survival rates for patients with bladder cancer in TCGA data set. The performance of nomograms in predicting overall survival rate was evaluated when compared to the AJCC stage. The performance of the prognostic nomograms was evaluated using Kaplan-Meier analysis and the AUC of the ROC curve. The Harel consistency score was adopted to obtain the discriminability of the nomination charts, and Kaplan-Meier analysis was adopted to plot the survival curves of the high-risk and low-risk groups.

### 2.7. Construction and Validation of Predictive Nomograms

To predict 1-, 2-, and 3-year overall survival of pancreatic cancer patients in TCGA data set, all independent prognostic characteristics and relevant clinical parameters were included in the building of a prognostic nomogram via a stepwise Cox regression model after testing for collinearity. The performance of the nomogram in predicting overall survival was confirmed using the AJCC stage as a control. The performance of the prognostic nomogram was evaluated using Kaplan-Meier analysis, AUC of the ROC curve, Harrell's concordance index, and a calibration plot comparing predicted and observed overall survival. Using a bootstrap method with 1,000 resamples, Harrell's concordance index was produced for the measurement of nomogram discrimination. The nomogram calibration curve was plotted for the evaluation of anticipated vs. observed overall survival. The patients were separated into three groups based on the total points of the nomogram and optimal cutoffs computed in X-Tile. Kaplan-Meier analysis was adopted to plot survival curves for the high-, medium-, and low-risk groups.

### 2.8. The Patient's Immune Microenvironment and Immunotherapy Are Correlated with the Signature of Phagocytosis Regulators

The ESTIMATE (estimating matrix and immune cells in malignant tumor tissue using expression data) program was adopted to obtain the matrix, immunity, and estimated scores (https://bioinformatics.mdanderson.org/public-software/estimate/) [31]. B, CD4^+^T, CD8^+^T, and dendritic cells; neutrophil; and macrophage abundance) Box and line diagrams depict the substantial difference in immunological scores between the high and low score groups, as well as the computed risk score and signature. The correlation analysis of phagocytosis regulators in bladder cancer by the TIMER (Tumor Immunity Estimation Resource) algorithm was conducted for evaluation (https://cistrome.shinvapps.io/TIMER/) [32]. The correlation analysis of phagocytosis regulators in the high-risk groups and the low-risk groups was estimated by TIMER and presented as a box diagram.

### 2.9. Analytical Statistics

R 3.4.3 and GraphPad Prism v.8.01 were used for statistical analysis (GraphPad Software, La Jolla, CA, USA). The 2 or Fisher's exact test was performed to examine categorical variables. In terms of paired samples, Student's *t*-test was performed to evaluate continuous variables. One-way ANOVA was adopted to examine multiple groups of continuous variables. Univariate and multivariate Cox regression models were used to evaluate survival. The hazard ratio (HR) and 95 percent confidence interval (CI) were adopted to find genes correlated with overall survival. *P* < 0.05 indicated a difference with statistical significance unless otherwise stated.

## 3. Results

### 3.1. MPOP Have a Role in Tumor Formation by Regulating Macrophage Phagocytosis

**Figure 1:**
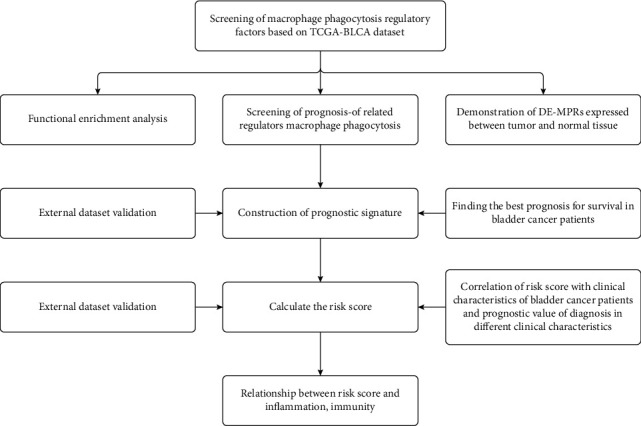
The flowchart illustrates the process of establishing the macrophage phagocytosis regulators (MPOP) signature and prognostic nomogram in bladder cancer of this study.

**Figure 2:**
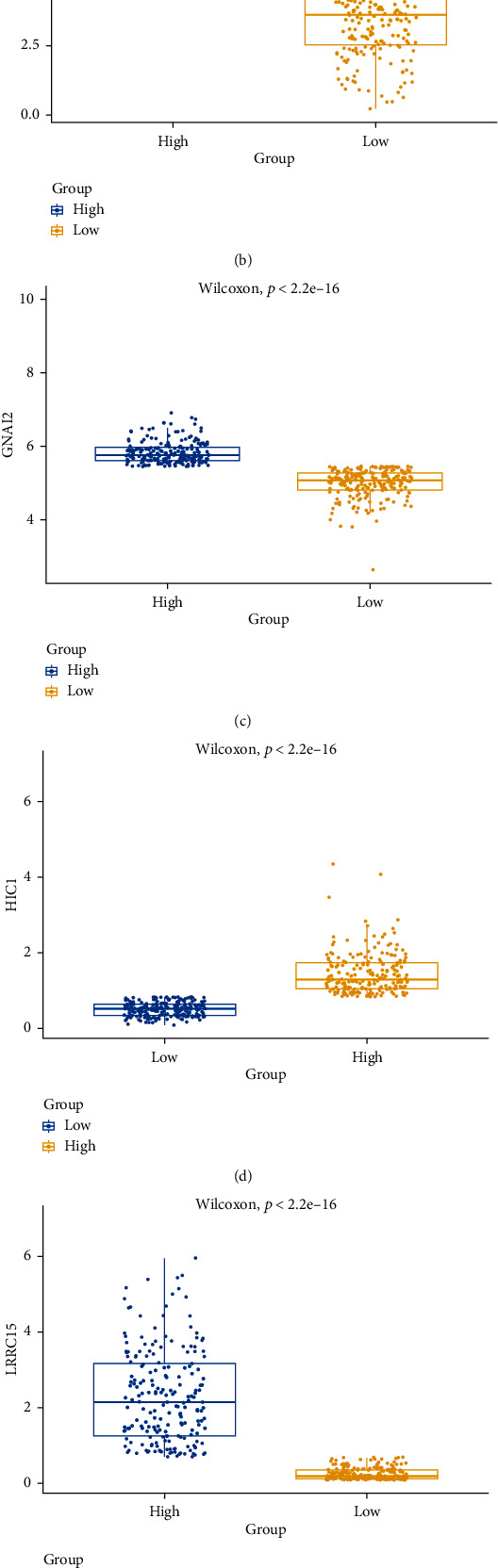
MPOP regulate the phagocytosis of macrophages. (a)–(f) demonstrate the differences in macrophage phagocytosis regulatory factor expression between groups, including AXL (a), BASBP1 (b), GNAV2 (c), HIC1 (d), LRRC15 (e), and RNF122 (f).

The work flow of this study is illustrated as Figure 1. To explore the mechanism of phagocytosis regulators in tumor development, we used ssGSEA to obtain the sample macrophage [24, 25] enrichment score and obtained the Pearson correlation between enrichment score and phagocytosis regulator expression, and the results showed that among 271 phagocytosis regulators, 88 gene expressions were correlated with macrophage enrichment score (Supplementary Figure 1, Supplementary Table 2-3). Then, to further explore the regulation of macrophage phagocytosis by MPOP, differences in macrophage phagocytosis regulatory factor expression and between-group differences were demonstrated by differential analysis (Figure 2). The above results show a regulatory role of MPOP on macrophage phagocytosis.

### 3.2. Functional Analysis of All Phagocytosis Regulators

**Figure 3:**
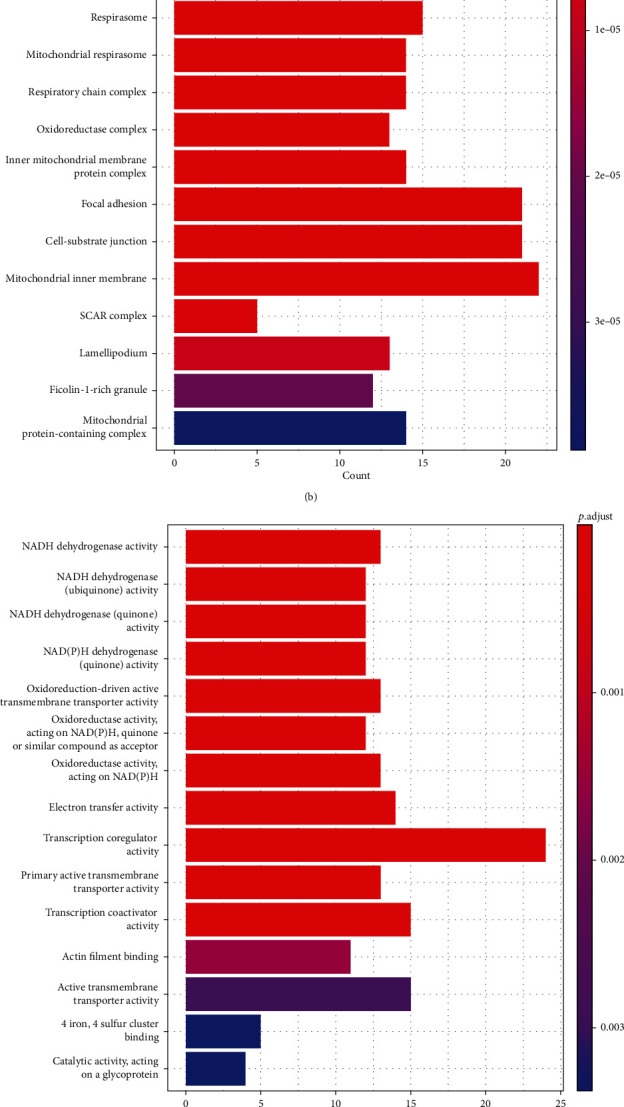
Functional analysis of macrophage phagocytosis regulatory factors: (a–c) enrichment analysis of macrophage phagocytosis regulatory factors BP, CC, and MF and (d) enrichment analysis of KEGG pathway, respectively.

The functions of DEGs were found using GO and KEGG pathway enrichment analysis (Supplementary Table 4-5). Biological processes correlated with mitochondrial oxidative respiratory chain, energy anabolism, and cell adhesions are considerably enriched in macrophage phagocytosis regulators. The findings are consistent with bladder cancer's hypermetabolic activity and extremely aggressive and metastatic characteristics (Figure 3(a)). The hypermetabolic activity of bladder tumors is a major important feature of their development. The biological processes significantly enriched also comprise mitochondrial respiration, respiratory chain NADH dehydrogenase complex, ATP synthesis, cell adhesion, cell adhesion and respiratory electron transport chain, protein complex, extracellular matrix organization, mitochondrial inner membrane, NADH dehydrogenase activity, and oxidoreductase activity and transcriptional activity of transmembrane transporters. Figures 3(b) and 3(c) present the enrichment analysis of cellular components and molecular functions. As revealed by KEGG pathway analysis, macrophage phagocytosis regulators play a certain role in fever, Alzheimer's disease, pathogenic E. coli infection, phagocytosis, oxidative phosphorylation, metastasis, and natural killer cell-mediated cellular immunity (Figure 3(d)). The above findings imply that macrophage phagocytosis regulators play a certain role in bladder cancer metabolism and may serve as a potential therapeutic method.

### 3.3. MPOP Regulators Are Widely Deregulated in Tumors

**Figure 4:**
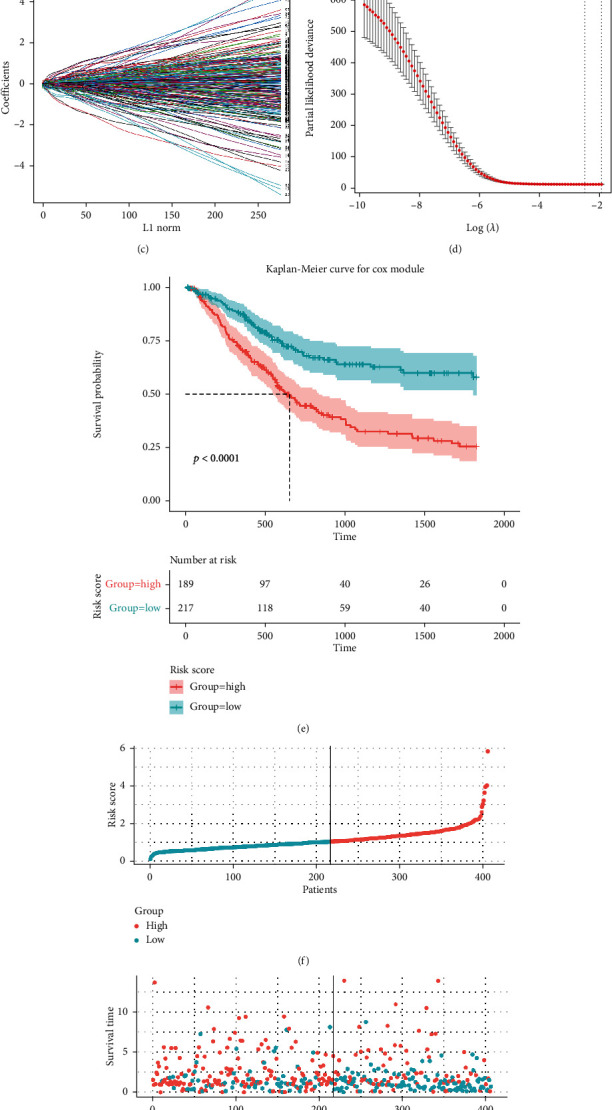
Identification and modeling of prognosis-associated macrophage phagocytosis regulators: (a) The result of PCA analysis demonstrating the efficacy of differentiating tumor from normal tissue. (b) Four phagocytic modulators screened out by COX univariate regression analysis with predictive value for the prognosis of tumor patients. (c, d) LASSO regression analysis modeling. (e) Demonstration of the predictive value of signature for 5-year survival in tumor patients. (f) Demonstration of the high value of signature in predicting 5-year survival. (f–h) Demonstration of the high-risk groups and the low-risk groups divided by (based on) 5-year survival, scores, and numbers of each group as well as the expression levels of survival time, survival events, and genes in relation to the grouping. (i) Demonstration of the high value of signature in predicting 5-year survival. (j) Columnar line graphs for the prediction of 1-year, 3-year, and 5-year overall survival in patients with bladder cancer.

To further understand the expression of macrophage phagocytosis regulators in bladder cancer and normal tissues, we analyzed 223 phagocytosis regulators and found that 138 phagocytosis regulators were extensively maladjusted in tumors and normal tissues (Supplementary Figure 2, Supplementary Table 6). The above findings imply that macrophage phagocytosis regulators may have a role in bladder cancer genesis and progression. Using PCA cluster analysis with normal sample data, we then demonstrated the ability of macrophage phagocytosis regulators to distinguish bladder cancer from normal bladder tissue (Figure 4(a)).

### 3.4. Identification of Prognostic-Associated MPOP Regulators and Construction of Prognostic Signature

**Figure 5:**
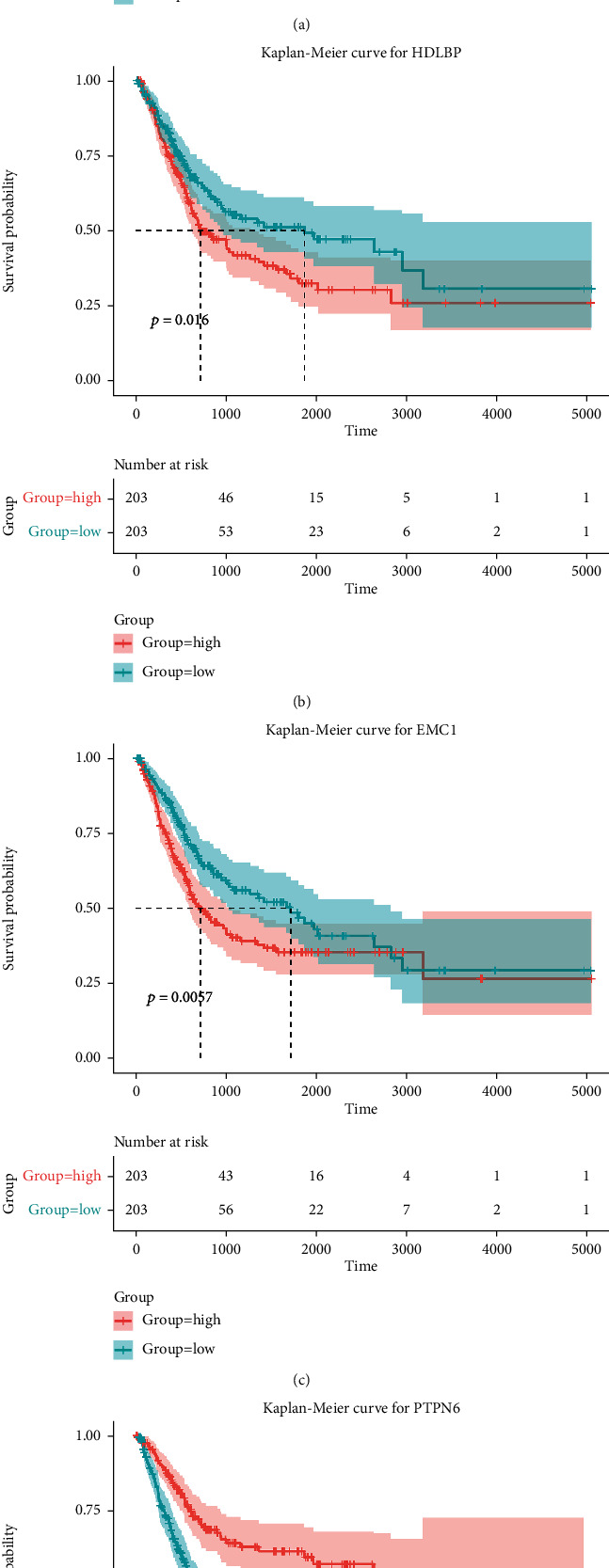
(a–d) show the univariate predictive value of the 4 genes for prognosis.

The following survival analysis comprised patients from TCGA BLCA data set. On the basis of LASSO regression analysis to identify four phagocytosis regulators related with bladder cancer prognosis, the baseline characteristics of the above patients are reported in Supplementary Table 1. Using a multivariate Cox analysis, we then created a prognostic signature (Figures 4(b)–4(d)). The above consists of HDLBP (High-Density Lipoprotein Binding Protein), EMC1 (ER Membrane Protein Complex Subunit 1), PTPN6 (Protein Tyrosine Phosphatase Non-Receptor Type 6), and IKAROS Family Zinc Finger 3 (IKZF3). The HR < 1 downregulation of PTPN6 and IKZF3 is considered a tumor suppressor, while the HR > 1 upregulation of HDLBP and EMC1 is considered an oncogene. Obtain the optimal cut-off value for the risk score using R program. In TCGA data set, patients were separated into two groups. The total survival rate was significantly better (*P* < 0.0001) in all groups with low-risk scores, according to the Kaplan-Meier survival curve (Figure 4(e)). The AUC of the risk score forecasts for 1-, 2-, and 3-year total survival was 0.690 (Figure 4(i)). This demonstrates that the prognosis model is accurate in its predictions. Surprisingly, we found that most of the macrophage phagocytosis regulators in our investigation were negatively linked with the risk model using thermography (Figures 4(f)–4(h)). The prognostic value of each gene is stable and accurate (Figure 4(j)), which can be applied to the clinical management of patients with BCa. In addition, four macrophage phagocytosis regulators related with prognosis of bladder cancer were analyzed. The findings revealed that the higher the HDLBP and EMC1 expressions, the worse the survival rate. The survival time was favorably linked with the expression of PTPN6 and IKZF3 (Figures 5(a)–5(d)). The above findings imply that the four DE-MPOP signatures are effective at predicting bladder cancer overall survival.

### 3.5. External Confirmation of MPOP Gene Signature Prognostic Characteristics

**Figure 6:**
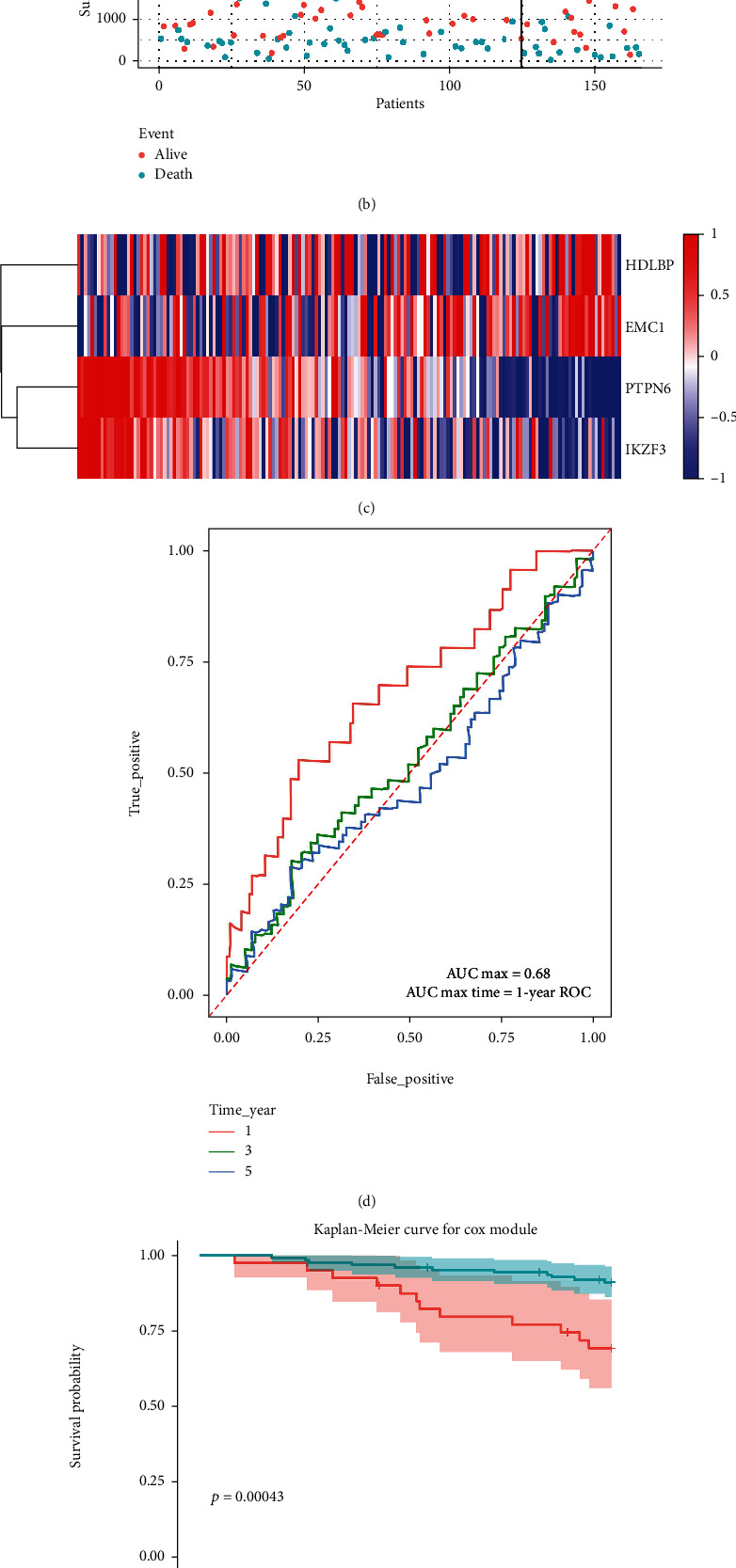
Validation of the predictive efficacy of the Signature in external data GSE13507: (a) shows the classification of the high-risk groups and the low-risk groups with 5-year survival as the boundary, together with the scores and numbers of each group. (b) shows the relationship between survival time, survival events, and grouping. (c) shows the expression levels of four macrophage phagocytosis regulatory factors in relation to grouping. (d) shows the validation of the Signature in the external data set, which was found to have equally high prognostic value in predicting 5-year survival. (e) shows the validation of the signature in an external data set for the prediction of 5-year survival of tumor patients.

**Figure 7:**
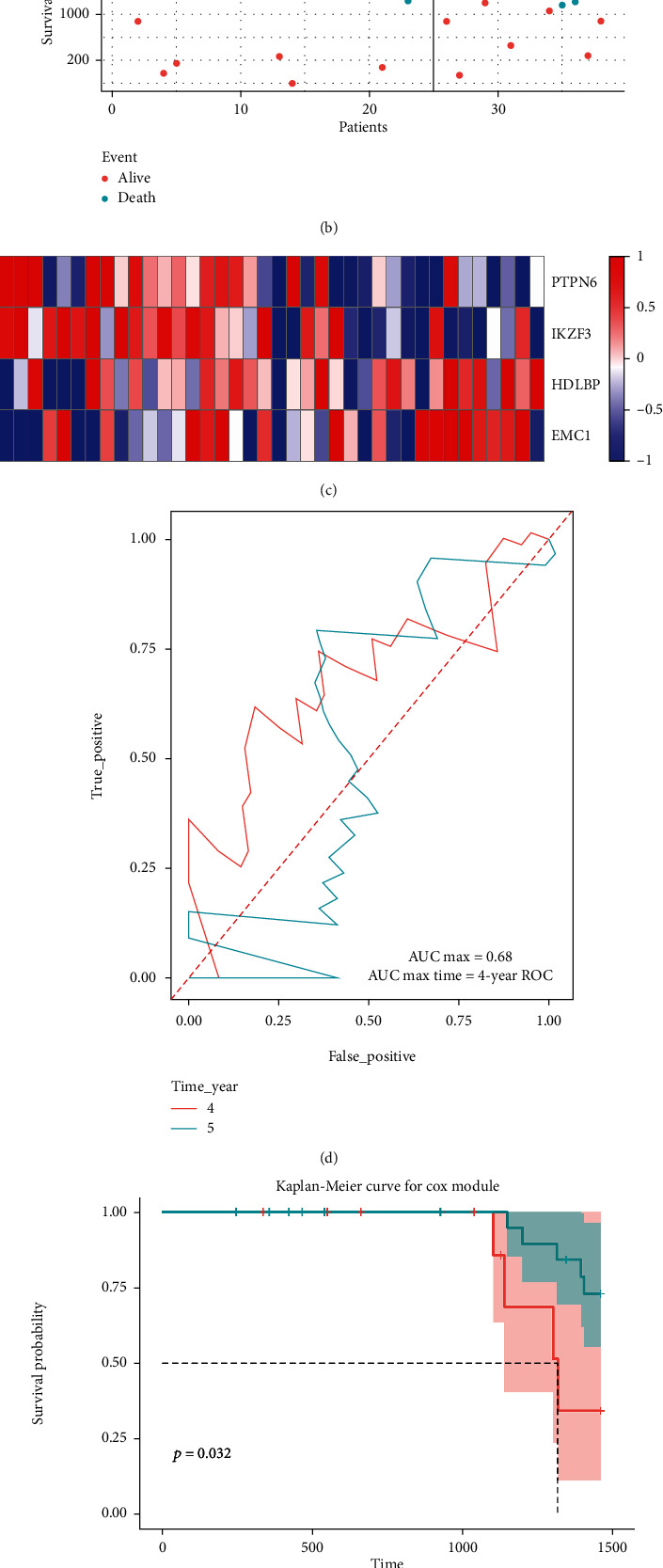
Validation of the predictive efficacy of Signature in external data GSE69795: (a) demonstrates the classification of the high-risk groups and the low-risk groups with 5-year survival as the boundary, together with the scores and numbers of each group. (b) demonstrates the relationship between survival time, survival events, and grouping. (c) demonstrates the expression levels of four macrophage phagocytosis regulatory factors in relation to grouping. (d) shows the validation of the signature in the external data set, which was found to have equally high prognostic value in predicting 5-year survival. (e) shows the validation of the signature in an external data set for the prediction of 5-year survival of tumor patients.

The four DE-MPOP predictive characteristics were validated using two external data sets, GSE13507 and GSE69795. Each patient's risk score was computed using the same formula. According to the optimal cutoff value obtained for each data set, patients were divided into two groups: low-risk and high-risk. In both data sets, Kaplan-Meier survival curves revealed a substantial difference in overall survival across the groups. The outcome in the high-risk group was much lower than that in the low-risk group (Figures 6(e) and 7(e)). Prognostic ability was then evaluated by the area under the transient ROC curve. Validation in the GSE13507 data set revealed that signature also had high prognostic predictive power in the external data set, and the highest predictive power for 1-year survival was found in this data set, reaching an accuracy of 0.68 (Figure 6(d)), while validation in the GSE69795 data set revealed the highest predictive power for 4-year survival, reaching an accuracy of 0.68 (Figure 7(d)). In the high-risk and low-risk categories, we found a favorable association between risk score and survival status (Figures 6(a), 6(b), 7(a), and 7(b)). The expression distribution of four DE-MPOP in high and low groups was shown based on the results of the risk heat map (Figures 6(c) and 7(c)). Overall, the external validation results indicate that MPOP characteristics are effective in predicting overall survival in patients with bladder cancer.

### 3.6. The Signature of Phagocytosis Regulators Is Correlated with Patient Prognostic Validation and Clinical Characteristics

**Figure 8:**
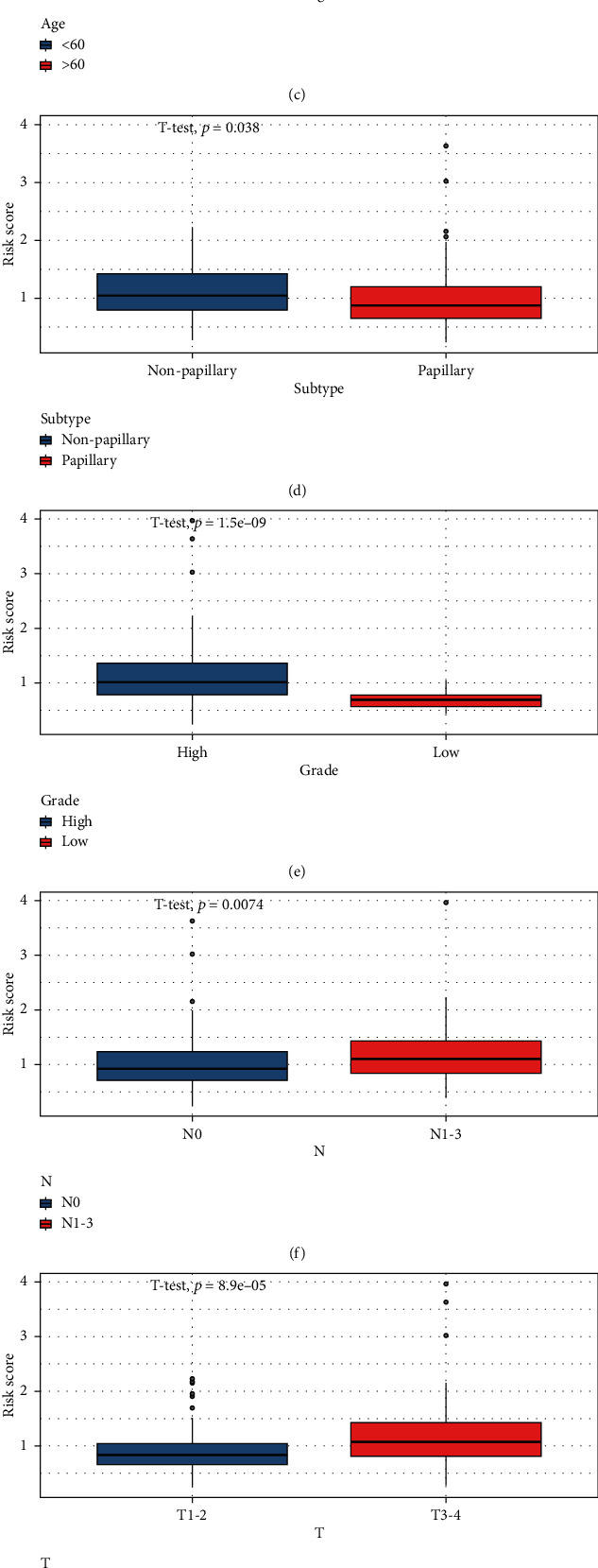
Correlation analysis between this signature and patients' clinical characteristics: (a, b) validation of the relationship between risk score and clinical characteristics by multifactor COX regression analysis in TCGA and GSE13507 data sets. (c–l) show the correlation analysis between high and low risk score groupings and clinical characteristics (age, subway, stage, grade, stage TMN, race, gender, tobacco years, and BMI).

The analysis included 418 patients from TCGA BLCA data set who gave the clinical information, such as age, gender, TCGA molecular typing, AJCC TNM stage, smoking history, and drinking history (Supplementary Table 1). Using univariate and multivariate cox regression analyses, prognostic markers for overall survival in bladder cancer were found. Multivariate Cox regression analysis confirmed that risk score and age were independent prognostic factors for patients with bladder cancer. However, similar results were obtained after validation of the external data of the GSE69795 and GSE13507 data sets (*P* < 0.05, Figures 8(a) and 8(b)). Next, to further understand the differences between risk score and patient clinical characteristics, we correlated the risk score with patient clinical characteristics (subtype, age, gender, grade, stage, AJCC TMN, race, BMI, and tobacco year), and the results showed that the risk score and risk score were significantly different from age, grade, subtype, stage, T, N, ethnicity, and smoking years, but not from gender and BMI groups (Figures 8(c)–8(l)). In addition, we also analyzed the prognostic efficacy of signature in different clinical characteristic groups and found that patients > 60 years of age, male patients, female patients, high grade, stages I-II, stages III-IV, M0, N0, N1-3, T1-2, T3-4, smoking time less than 1 year, and >1 year had prognostic significance (Supplementary Figure 3). The above results indicate that signature built from four DE-MPOP is an independent prognostic factor for patients with bladder cancer, which is significantly correlated with clinical characteristics of patients with bladder cancer.

### 3.7. The Signature of Phagocytosis Regulators Is Correlated with the Patient's Immune Microenvironment and Immunotherapy

**Figure 9:**
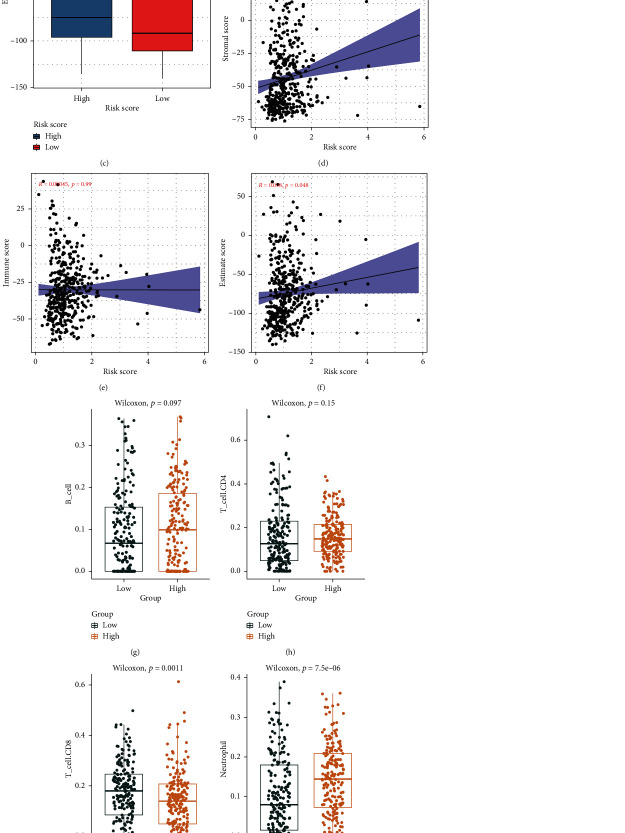
Signature correlation with immune score and immune infiltrating cells: immune score vs. risk score correlation analysis; (a–c) showing differences between immune scores in the high-risk groups and the low-risk groups; (d–f) showing immune score, stromal score, and estimate score in correlation with risk score; (g–l) showing phagocytosis regulatory factors obtained by TIMER immune infiltration results.

This paper looked at the tumor immune correlation of DE-MPOP signature, evaluating the correlation between gene characteristics and tumor purity, as well as the presence of infiltrating stroma/immune cells in tumor tissue, to see if there was any link between risk score, immune score, and estimated score. The ESTIMATE algorithm was adopted to obtain stromal, immune, and estimate scores using expression data acquired from TCGA BLCA data set. Substrate and estimated scores were comparable between the homogeneous and low-risk groups. The immunological score, on the other hand, was significantly higher in the high-risk group, indicating that there was greater immune cell infiltration in the tumor tissue (Figures 9(a)–9(c)). In addition, our results also suggested an excellent positive correlation between the risk score and the matrix score (*R* = 0.16, *P* < 0.001, Figure 9(d)), as well as estimated scores (*R* = 0.098, *P* < 0.05, Figure 9(f)). Next, the TIMER algorithm was adopted to further estimate macrophages. The infiltration abundance of B lymphocytes, CD4 T lymphocytes, CD8 T lymphocytes, and dendritic cells was analyzed, and their correlation at high and low risk was analyzed. The analysis results revealed that in T cell, CD8 was significantly different from neutrophil (Figures 9(g)–9(l)). Lastly, we also analyzed the correlation analysis of signature with immune checkpoints and proinflammatory factors. Surprisingly, the results showed that the genes contained in signature were significantly correlated with immune checkpoints and inflammatory factors such as CTLA4, IL18, and IL6 (Supplementary Figure 4A-I). Meanwhile, we also found a significant correlation between risk score and the expression level of IL6 (Supplementary Figure 4J). The above results confirm that signature built from four DE-MPOP may be significantly correlated with immune infiltration of bladder cancer and tumor immunotherapy and may be a new potential target for immunotherapy in patients with bladder cancer.

## 4. Discussion

BCa has been found as the most frequent cancer of the urinary tract, which is characterized by a complex biological activity, a high recurrence rate, and a high incidence of metastatic spread **[**33**]**. Immunotherapy appears to be the most promising of the current BC treatments **[**34**]**. BCG was licensed for immunotherapy of BC in 1990 and has had a high rate of success, although it should be noted that roughly 40% of BC patients do not react to BCG and 15% of BC patients progress to MIBC after treatment **[**35, 36**]**. Tumor cells are capable of eluding immune responses by changing immunological checkpoints in accordance with recent discoveries **[**37, 38**]**. As a result, current research on ICI for immunological escape prevention is attracting a lot of attention **[**39–41**]**. The U.S. Food and Drug Administration (FDA) approved 5 species of IC, pembrolizumab, nivolumab, atezolizumab, durvalumab, and avelumab for the treatment of advanced and metastatic disease BC [42]. However, the mechanism of immunotherapy in patients with bladder cancer has not been clarified. Monoclonal antibody therapy has been presented in cancer therapy “Magic bullet,” since in principle they are capable of tailoring the way cancer cells are destroyed by macrophages, which are abundant in tumor tissue. However, the mechanisms by which cancer cells escape phagocytosis remain unclear. Accordingly, this paper focused on tumors—functional genomic approaches to macrophage interactions. In order to explore the mechanism of interaction between bladder tumor macrophages, 223 macrophage phagocytic regulatory factors have been identified by general gene sequencing. The expression of 88% MPOP in bladder cancer tissue was significantly different from that in normal bladder tissue. DE-MPOP have been identified. It could play a role in the occurrence and progression of bladder tumors. Functional enrichment analysis showed that MPOP was associated with bladder cancer, and high metabolic activity was closely related to high invasion and metastasis. Analysis showed that MPOP was involved in related pathways such as fever, Alzheimer's disease, pathogenic Escherichia coli infection, phagocytosis, oxidative phosphorylation, metastasis, and natural killer cell-mediated cellular immunity. The above results indicate that MPOP It may be involved in the metabolism of bladder cancer and the occurrence and development of inflammatory immune response, thus affecting the exemption of bladder tumors from treatment.

Pass Lasso-Cox Regression builds a prediction Bladder Novel method for overall cancer survival MPOP Features. PTPN6 and IKZF3 were downregulated and found as a protective factor. HDLBP and EMC1 were upregulated in tumor tissue and correlated with poor survival. This MPOP features are bladder-independent prognostic factors for cancer. In the low-risk group, the prognosis of the patients was significantly higher than that of the high-risk group. In TCGA data sets and external data sets, MPOP Prognostic performance of features GSE13057 and GSE69795 was validated. AUC stands for Area Under the Curve. For annual overall survival, the predictions of 1 year, 3 years, and 5 years were confirmed. For MPOP, it exhibits a high level of predictability. Nomograms have become frequently utilized in clinical oncology to estimate prognosis in recent years [43]. They can consider various prognostic factors (e.g., molecular and clinicopathological factors). A relatively simple output can be employed to obtain and illustrate the numerical probability of a clinical occurrence. Row and column line drawings, as compared to standard staging, can induce MPOP prognosis prediction and facilitate clinical decision-making and individualized treatment. Accordingly, we established the model correlated with bladder cancer prognosis MPOP and construction of clinical pathology parameters and accurately predict the overall survival. Further analysis also found that risk score and age, grade, subtype, stage, T, N, ethnicity, and years of smoking were significantly different, compared with gender and BMI. The groups were not significantly different. Furthermore, this signature was prognostic in 60 years, male patient, female patients, high grade, stages I-II, stages III-IV, M0, N0, N1-3, T1-2, T3-4, smoking duration less than 1 year, and >1. The prognosis of patients is predictive. The above findings reveal that MPOP signatures and nomograms can be adopted to predict tumor development and overall survival in patients with bladder cancer.

Paired MPOP for further analysis of the MPOP genes revealed that two of the four genes in the MPOP signature have been shown to be widely expressed in a variety of tumors, where ER Membrane protein complex 1 (EMC1) is an endoplasmic reticulum membrane protein complex, one of the family members. The synthesis, folding, and modification of proteins in the endoplasmic reticulum, as the principal bearer of life functions, are carefully regulated processes that can affect the function, fate, and survival of cells. In the case of ER homeostasis, all of the above mechanisms must be established. ER stress has been correlated with the development of a variety of cancers in a number of studies [44, 45]. The study confirmed that the ER membrane protein complex (EMC), as a member of EMC1, promotes viral transport in the ER and is associated with the body's infection mechanism [46]. The current study confirm that ECM1 produced by hepatocytes inhibits TGF*β* activation to prevent liver fibrosis in mice [47]. The other gene, PTPN6 (protein tyrosine phosphatase non-receptor type 6), is a tyrosine phosphatase that affects a variety of cellular processes, including cell proliferation, differentiation, the mitotic cycle, and neoplastic transformation. PTPN6 refers to a tyrosine phosphatase capable of dephosphorylating oncogenic kinases, and it has been identified in nonreceptor proteins. PTPN6 has been shown to be a tumor suppressor [48]. Existing research found that PTPN6 is correlated with cancers like hepatocellular cancer, renal cell cancer, and gastric cancer's prognosis and progression [49–51]. Another recent study reported that PTPN6 is capable of decreasing prostate cancer cell proliferation and enhancing apoptosis, thus revealing that it can be a potential therapeutic target for prostate cancer [52]. PTPN6 could be a new prognostic biomarker, according to Shen et al. (TCGA database determination) [53]. According to Wen et al., PTPN6 has been shown to block JAK/STAT, NF-B, and AKT signaling pathway activation in hepatocellular cancer progression [49]. The above studies have confirmed that PTPN6 may be a novel potential biomarker affecting tumor development. Nevertheless, the findings of this investigation revealed that PTPN6 expression was dramatically downregulated in individuals with bladder cancer. Its specific mechanism of action in bladder cancer has not been elucidated. Interestingly, it has been recently documented that myeloid Ets-2 Loss attenuation is given by Ptpn6 point mutation IL-1*α*-mediated inflammatory disease [54]. PTPN6 of expression for VitD3-mediated inhibition of macrophage foam cell formation by autophagy [55], according to the research by Kumar et al.. In addition, several recent researches have verified this. PTPN6 has also been demonstrated to have a variety of roles in immune cells, including neutrophil survival time, B cell activation, and T cell activation and survival time [56–59]. The above findings suggest that PTPN6 may be one that affects the progression of the disease by regulating changes in macrophages and inflammatory factors in inflammatory diseases. An existing study showed that PTPN6 plays an important role in antitumor immunity [60]. Watson et al. reported PTPN6-specific mechanisms of action in tumor immunity, for additional research. The potential of cells to suppress tumor growth can be enhanced by adoptively transferring CD8^+^ T cells, according to study results [61]. PTPN6 can also increase the effect of chemotherapeutic drugs by binding to blocking antibodies in cancer immunotherapy [48, 62]. Surprisingly, the results of experimental studies by Chen et al. similarly confirmed that PTPN6 is a prognostic gene in bladder cancer [63]. This research suggests that PTPN6 may play an important role in bladder tumor immunotherapy. However, the mechanism of action in bladder cancers of IKZF3 and density lipoprotein-binding protein (HDLbp), which has not been reported, may be a new prospective therapeutic target for immunotherapy in bladder patients and is worthy of further exploration.

Paired MPOP the mechanism of action of related gene-regulated macrophages in cancer immunotherapy continues to be further analyzed. In this paper, further functional analysis of macrophage phagocytosis regulators revealed that most of the genes were enriched in mitochondrial oxidative respiratory chain, cell adhesion, and glucose metabolism KEGG. The results showed that most of the macrophage phagocytosis regulators were mainly enriched in inflammatory diseases, macrophage phagocytosis, and immune response. Multiple studies have recently used it. Inflammatory cell density and stroma infiltrating within tumor tissue were evaluated by immunoassaying and stromal scoring and density of cell [31]. In other forms of malignancies, they have been correlated with a poor clinical outcome [64]. This paper's findings of substrate scores were obtained as the total of low-risk and comparable groups, and they were positively correlated with risk scores. Immune scores, on the other hand, were significantly higher in the high-risk group, indicating that immune cells infiltrate tumor tissue in greater numbers. In addition, this paper found significant differences between MPOP in CD8^+^ T cells and neutrophil. Surprisingly, the results of this paper also revealed that the genes contained in signature were significantly correlated with immune checkpoints and proinflammatory factors such as CTLA4, IL18, and IL6. The levels of IL6 expression and the simultaneous risk score were strongly associated.

Macrophages play a role in local and systemic inflammation, as well as tissue remodeling and immunology. They perform a variety of tasks, including phagocytosis, antigen presentation, microbial cytotoxicity defense, and cytokine and complement component secretion [8]. Tumor-associated macrophage (TAM) is an important component of the tumor microenvironment involved in cancer progression and metastasis. It is widespread in various tumors **[**65**]**. They are closely involved in angiogenesis and immunosuppression of normal and malignant tissues, as well as profibrotic activities [66]. TAM may encourage tumor development, invasion, metastasis, and medication resistance [67]. TAM initiation, angiogenesis, metastasis, treatment resistance, and antitumor immunosuppression are all investigated for its tumor-promoting activity [68]. According to the standard notion, there are two polarization activation phases in macrophages: classical (M1, by lipopolysaccharide and IFN-induction) as well as replacement (M2, by IL-4 or IL-13 induction) [69]. Previous studies have found that TAM mainly has an M2-like phenotype, manifested as immunosuppression and tumor promoting progression (68). Therefore, targeting M2-like TAMs to deplete them in TME or reverse m2-like TAMs to an M1-like phenotype directly enhances their cytotoxicity. Indirect stimulation of cytotoxic T cells to eliminate tumor cells is a potential strategy for anti-tumor immunotherapy [70, 71]. TAM has been clarified. There are numerous mechanisms underlying the function in tumor immunosuppression, and clinical trials of potential therapeutic drugs based on the above novel targets have recently been done [71, 72]. Furthermore, some recent studies have focused on phagocytosis, macrophages' fundamental function, by targeting the CD47/SIRP reactivation of antitumor immunity [73], PD-1/PD-L1 [74], and CD24/Siglec-10 [75] routes. All of the above tactics will be successful. It has pushed cancer immunotherapy to the forefront.

The choice of macrophage phagocytosis regulators of expression levels is the basis of our predictive model. This method is less expensive and more clinically useful than whole genome sequencing. Clinicians may be capable of making the prognosis of specific patients using our nomogram, which combines MPOP characteristics and clinicopathological factors. Its graphical grading system is simple to grasp, which makes it simple to tailor treatment and make medical decisions. To the best of our knowledge, the one presented in this paper. MPOP prognostic characteristics and nomograms based on it have not been previously reported. Accordingly, it may be the bladder cancer immunotherapeutic molecular mechanisms, and prognosis prediction provides new insights. Furthermore, the DE-MPOP comprehensive analysis from various data sets with a high degree of dependability was acquired in this work. Prior to this research, four of the genes in the signature have not been correlated with bladder cancer. The above DE-MPOP may be facing prospective molecular targets in bladder cancer immunotherapy.

There are a number of limitations to this paper as well. First, TCGA retrospective data construction database was adopted to create our prognostic model. In DE-MPOP signature, some cell investigations and animal tests should be performed independently or in combination. The experiment was carried out to test the model's forecast accuracy and to obtain the underlying process. Second, it is necessary to validate its clinical applicability using prospective real-world data. Third, the use of only a single marker to build a prognostic model is flawed because many of the important prognostic genes in may have been excluded. Finally, the link between immune status and risk score has not been experimentally validated.

## 5. Conclusion

In brief, we built an inclusion of four prognostic models of DE-MPOP, which were shown to have independent correlation with OS and can accurately predict BLCA prognosis of immunotherapy. Comprehending the underlying mechanism and significance of the above DE-MPOP in BLCA Immunotherapy can provide insights for the determination of BLCA immunotherapy targets.

## Data Availability

All generated as well as analyzed data in the present research are contained in this manuscript.

## Supplementary Materials

Supplementary 1 Supplementary Figure 1: the picture shows the correlation analysis between the expression and enrichment scores of MPOP in TCGA data set.

Supplementary 2 Supplementary Figure 2: MPOP are extensively dysregulated in tumors: The pictures demonstrate the changes in the differential expression of MPOP between tumor tissues and normal tissues in bladder cancer.

Supplementary 3 Supplementary Figure 3: prognostic efficacy analysis of signature in different clinical characteristics groups:

Supplementary 4 Supplementary Figure 4: correlation analysis of risk score; prognosis-associated macrophage phagocytosis regulatory factors with immune checkpoints and proinflammatory factors; A-I plot showing the correlation between immune checkpoints and anti-inflammatory factors and risk score; figure J showing the IL-6 differences between the high-risk and low-risk groups.

Supplementary 5 Supplementary Table 1: characteristics of patients in TCGA data sets.

Supplementary 6 Supplementary Table 2: list of 223 regulators of macrophage phagocytosis.

Supplementary 7 Supplementary Table 3: list of 88 MPOP correlated with macrophage enrichment fraction.

Supplementary 8 Supplementary Table 4: functional enrichment analysis of the MPOP: the list of GO enrichment analysis of MPOP.

Supplementary 9 Supplementary Table 5: the list of enriched KEGG pathways of the MPOP.

Supplementary 10 Supplementary Table 6: the list of MRPs differentially expressed in bladder cancer.
