# Supplementary figures and images for: Prognostic Signature Development on the Basis of Macrophage Phagocytosis-Mediated Oxidative Phosphorylation in Bladder Cancer
Source: Oxid Med Cell Longev. 2022 Sep 29;2022:4754935. doi: 10.1155/2022/4754935 (PMC9537622; doi:10.1155/2022/4754935)

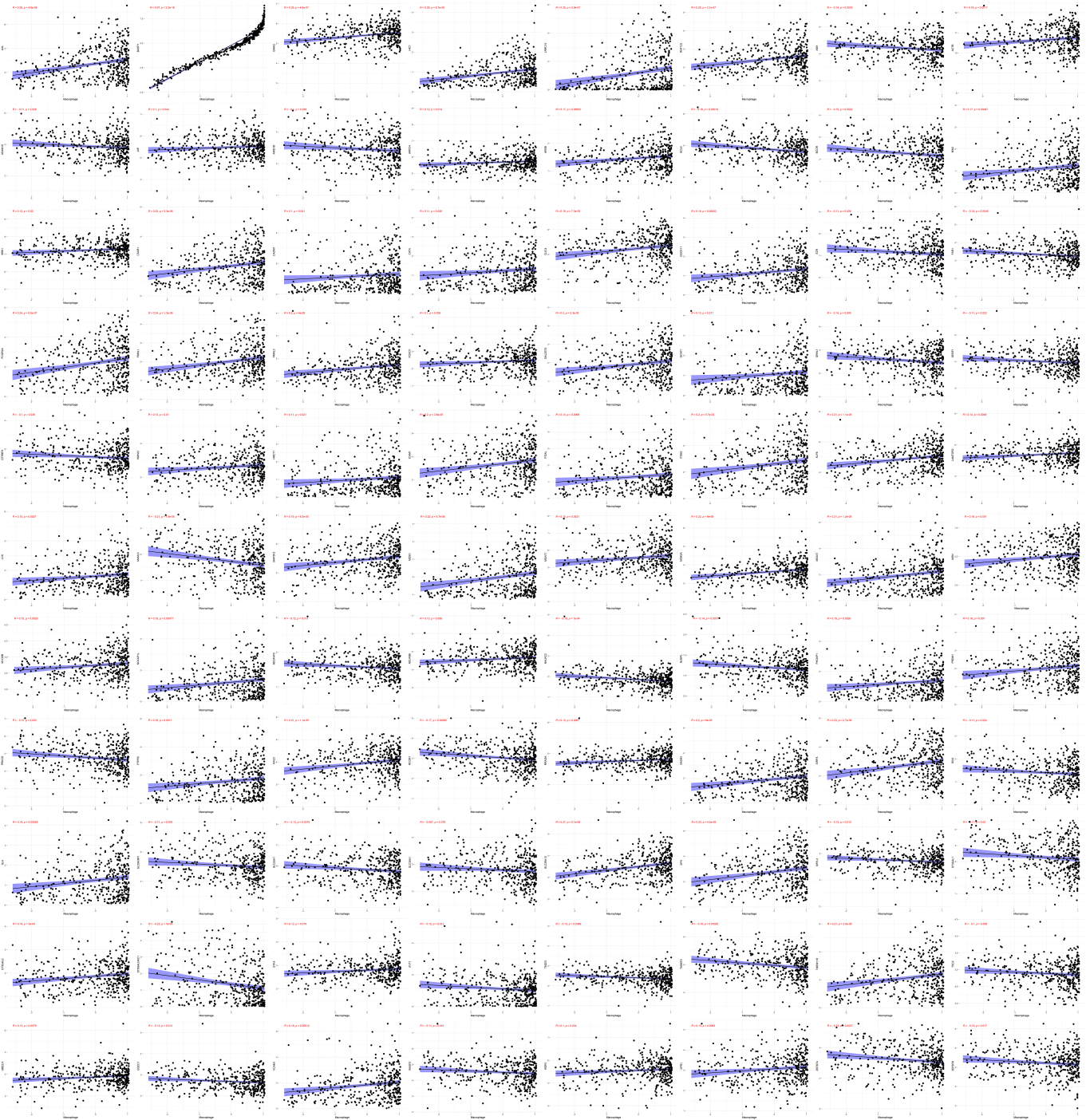

Supplement: Supplementary 1 — Supplementary Figure 1: the picture shows the correlation analysis between the expression and enrichment scores of MPOP in TCGA data set. [file 4754935.f1.pdf]

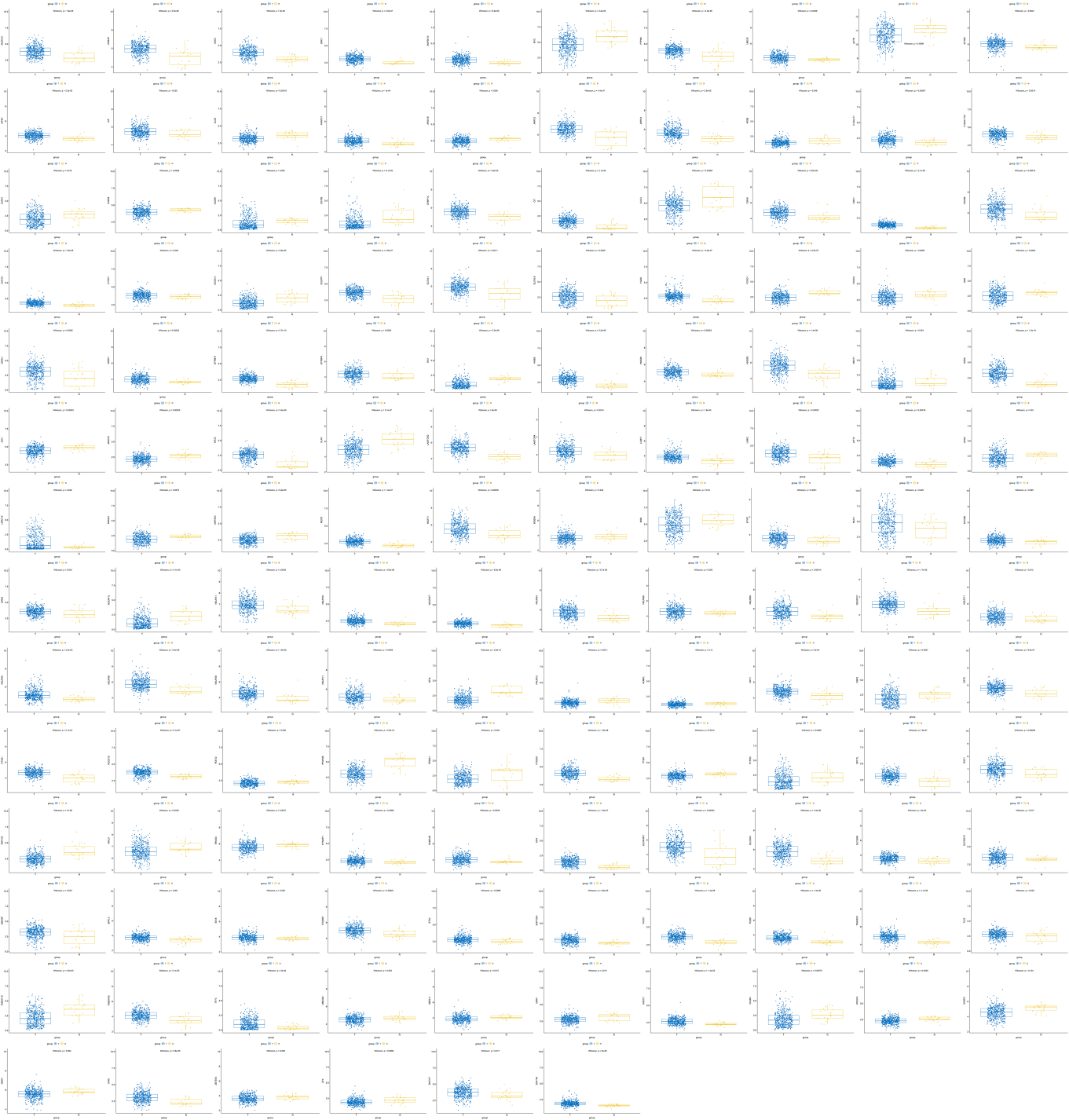

Supplement: Supplementary 2 — Supplementary Figure 2: MPOP are extensively dysregulated in tumors: The pictures demonstrate the changes in the differential expression of MPOP between tumor tissues and normal tissues in bladder cancer. [file 4754935.f2.pdf]

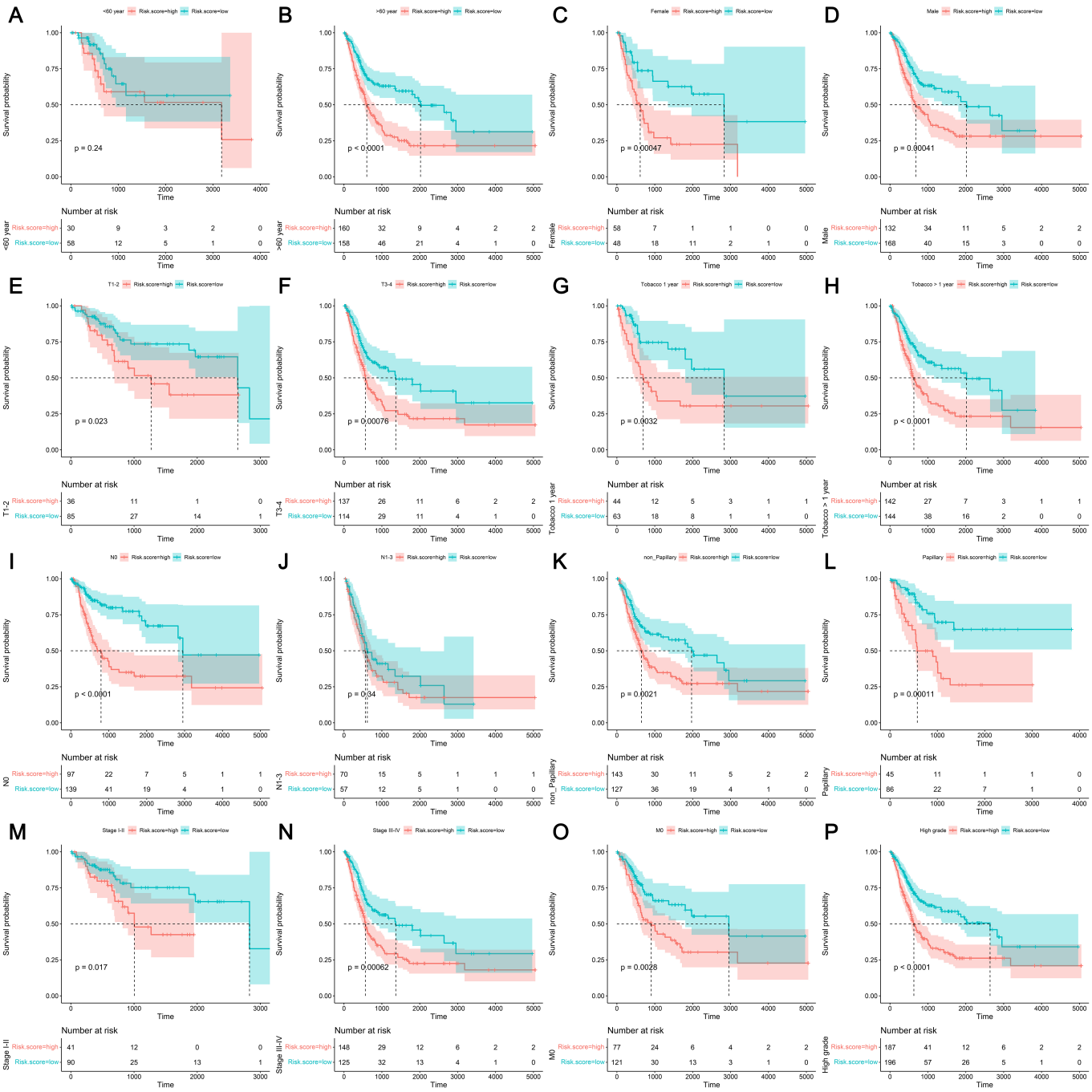

Supplement: Supplementary 3 — Supplementary Figure 3: prognostic efficacy analysis of signature in different clinical characteristics groups: [file 4754935.f3.pdf]

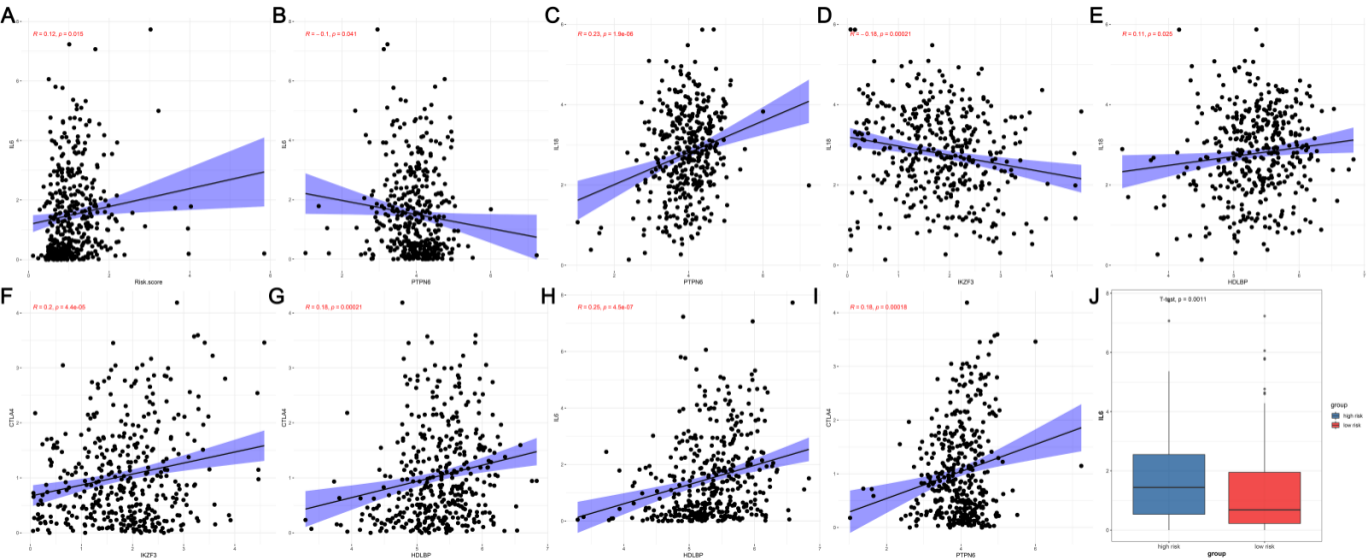

Supplement: Supplementary 4 — Supplementary Figure 4: correlation analysis of risk score; prognosis-associated macrophage phagocytosis regulatory factors with immune checkpoints and proinflammatory factors; A-I plot showing the correlation between immune checkpoints and anti-inflammatory factors and risk score; figure J showing the IL-6 differences between the high-risk and low-risk groups. [file 4754935.f4.pdf]
