# Supplementary material for: Prognostic Signature Development on the Basis of Macrophage Phagocytosis-Mediated Oxidative Phosphorylation in Bladder Cancer
Source: Oxid Med Cell Longev. 2022 Sep 29;2022:4754935. doi: 10.1155/2022/4754935 (PMC9537622; doi:10.1155/2022/4754935)
Supplement: Supplementary 5 — Supplementary Table 1: characteristics of patients in TCGA data sets. [file 4754935.f5.pdf]

TCGA and GEO database bladder cancer expression profiling dataset

Dataset ID	Platform	Bladder cancer	Normal
TCGA	-	418	38
GSE13507	GPL6102	246	10
GSE69795	GPL6244	61	0

Basic information of tumor patients in TCGA dataset

Characteristic		Number
Gender	Male	308
	Female	109
Age	>60	326
	<60	91
Subtype	Papillary	135
	Non-Papillary	277
Grade	Low	21
	High	393
M	M0	200
	M1	11
	MX	203
N	N0	241
	N1-3	133
	NX	37
T	T1-2	125
	T3-4	258
Stage	I	3
	II	131
	III	143
	IV	138
Tobacco	>1 year	293
	<1 year	110
