# Supplementary material for: Prognostic Signature Development on the Basis of Macrophage Phagocytosis-Mediated Oxidative Phosphorylation in Bladder Cancer
Source: Oxid Med Cell Longev. 2022 Sep 29;2022:4754935. doi: 10.1155/2022/4754935 (PMC9537622; doi:10.1155/2022/4754935)
Supplement: Supplementary 6 — Supplementary Table 2: list of 223 regulators of macrophage phagocytosis. [file 4754935.f6.pdf]

List of 223 regulators of macrophage phagocytosis
CD47
SIRPA
PTPN6
PTPN11
MYC
FCGR2A
GNE
CMAS
NANS
C1GALT1C1
QPCTL
SLC35A1
MS4A1
CAB39
UBE2D3
ARID1A
PDCD10
C1GALT1
PTEN
APMAP
RTN4IP1
AIFM1
FDX1
NDUFA1
GTPBP6
NDUFS8
SMARCC1
TACO1
CMC1
ATP5SL
SLC39A9
SS18
CHMP1A
GRSF1
C17ORF89
NDUFAF7
PDE12
UQCC1
NDUFAF5
HIGD2A
NDUFB9
MECR
WDR1
COX18
RHOH
HMGB1
SLC25A1
NDUFS6
FOXO1
TMEM261
HMGB2

CS
POU2F2
ADAM10
NDUFB6
MTIF3
MTO1
UBR4
NDUFB4
LIPT2
HMHA1
YBEY
ALAD
NXT1
OTUB1
NDUFV1
GTPBP3
NUBPL
NDUFS2
NDUFB11
LDB1
SAMD4B
C1ORF233
ZBTB7A
TIMMDC1
STK4
AP000721.4
STARD7
NFIA
UBE2K
VPS37A
NDUFA9
NDUFA8
ELOVL1
COX5B
PTPRC
NDUFAF3
ACTB
HMBS
NDUFC1
ARID1B
SPI1
TMEM38B
NDUFS7
PRKCD
GFI1
SMAGP
MUC21
ST6GALNAC1
ITGB2
OSR2
MUC1
CD1C

GAL3ST4
FUT6
ST3GAL1
LRRC15
TLE3
PRDM1
SPN
MUC12
HDAC9
ELOVL6
GFI1B
IRX5
MS4A7
MS4A14
C5AR1
CD44
IQGAP2
CBFA2T3
JMJD1C
CD38
ALCAM
PPAP2B
FCGR2B
PODXL
HIC1
BCL9L
MAML2
SPIB
CLIC4
SLA
PIK3AP1
FAR1
MAML1
POU2AF1
ZEB2
SASH3
SLC9A3R1
DOCK11
HES7
BCOR
TSPAN15
GAL3ST2
RAC2
FOXO4
AXL
LIMK2
SLC39A13
CADM1
CAPN6
MAML3
CLDN18
ST3GAL2

VSIG8
IKZF3
CEBPE
SYK
FMNL3
MAP3K3
ICAM1
EZR
FCGR1B
LCK
FMNL1
GPR114
GRHL1
PNMA5
ZC3HAV1
VCAM1
ZNF746
BTK
FAM81A
SLA2
ZNF683
CD79B
CIITA
PPP3CA
ZBTB7B
BCL6
MSN
IER5L
TCEB1
MAK
MAP3K10
ZNF311
MOB3A
FCRL3
ZDBF2
LCA5L
CIT
GCNT1
TMEM119
SIX4
SUV420H1
GYPA
ISL2
ZFX
TP73
SYT1
MEX3B
RNF122
LPIN2
MED13
NHLRC2
ACTR2

NCKAP1L
SUPT20H
ACTR3
ABI1
DOCK2
CYFIP1
ARPC2
ARPC4-TTLL3
FLII
ARPC4
SPPL3
LCMT1
RAC1
ARPC3
MAPK1
AMBRA1
MYO9B
RPL21
AIP
KIF23
OSTC
FADD
ANAPC7
JAK1
HDLBP
WASF2
RRAGA
KLF6
TM2D1
EMC1
TMEM165
BIN2
MGAT1
BRK1
LAMTOR4
RP11-45M22.4
TM2D2
BRD2
USP22
XPR1
PTDSS1
LAMTOR2
ZNF217
PIK3R5
ITGAL
TSC2
CDK2
OTUD5
SPTSSA
TM9SF3
UBE2J1
ARHGDIG

LCE1A
ARSB
NUP153
CXCL6
DOLPP1
HRC
RCOR1
SPTLC2
MIB2
USP34
GNAI2
BASP1
