# Supplementary material for: Prognostic Signature Development on the Basis of Macrophage Phagocytosis-Mediated Oxidative Phosphorylation in Bladder Cancer
Source: Oxid Med Cell Longev. 2022 Sep 29;2022:4754935. doi: 10.1155/2022/4754935 (PMC9537622; doi:10.1155/2022/4754935)
Supplement: Supplementary 7 — Supplementary Table 3: list of 88 MPOP correlated with macrophage enrichment fraction. [file 4754935.f7.pdf]

gene	R	P value
AXL	0.28	4.9E-09
BASP	0.97	2.2E-16
GNAI2	0.25	0.00000046
HIC1	0.28	5.7E-09
LRRC15	0.25	0.00000039
RNF122	0.25	0.00000031
ABI1	-0.14	0.0035
ACTB	0.16	0.0011
ADAM10	-0.11	0.026
AIP	0.1	0.044
ARID1B	-0.1	0.039
ARPC4	0.12	0.014
ARSB	0.17	0.00052
BCL6	-0.18	0.00019
BCOR	-0.15	0.0022
BIN2	0.17	0.00047
BRK1	0.12	0.02
C5AR1	0.22	0.0000053
CADM1	0.1	0.041
CIITA	0.11	0.028
CLIC4	0.19	0.000073
DOCK11	0.18	0.00022
EZR	-0.11	0.026
FAR1	-0.14	0.0048
FCGR2A	0.24	0.00000089
FMNL1	0.24	0.0000013
FMNL3	0.2	0.00004
FOXO1	0.1	0.038
GAL3ST4	0.2	0.000053
GCNT1	0.13	0.011
GRHL1	-0.14	0.005
GRSF1	-0.11	0.022
GTPBP3	-0.1	0.036
HMHA1	0.13	0.01
HRCT1	0.11	0.021
ICAM1	0.2	0.000038
ITGAL	0.13	0.0069
ITGB2	0.2	0.000057
KLF6	0.21	0.000011
LAMTOR2	0.14	0.0048
LCK	0.15	0.0027
MAML3	-0.21	0.000018
MAP3K3	0.19	0.000083
MDSC	0.22	0.0000057
MGAT1	0.15	0.0021
MOB3A	0.22	0.000009

MS4A7	0.21	0.000012
MSN	0.16	0.001
MYO9B	0.15	0.0025
NCKAP1L	0.18	0.00017
NDUFAF5	-0.12	0.013
NDUFB6	0.13	0.008
NHLRC2	-0.19	0.0001
NUBPL	-0.14	0.0037
PIK3AP1	0.15	0.0026
PRDM1	0.16	0.001
PRKCD	-0.15	0.003
PTPRC	0.16	0.0011
RAC2	0.21	0.000011
RCOR1	-0.17	0.00065
RRAGA	0.13	0.0067
SASH3	0.2	0.00006
SIRPA	0.23	0.0000027
SIX4	-0.11	0.0024
SLA	0.18	0.00025
SLC9A3R1	-0.11	0.029
SLC25A1	-0.13	0.0075
SLC35A1	-0.087	0.078
SLC39A13	0.27	0.000000021
SPI1	0.22	0.0000045
SPPL3	-0.12	0.012
ST3GAL1	-0.11	0.02
ST3GAL2	0.18	0.0003
ST6GALNAC	-0.22	0.00001
STK4	0.12	0.019
SYT1	-0.12	0.012
TM2D1	-0.13	0.0089
TM9SF3	-0.18	0.00025
TMEM119	0.21	0.000026
TSC2	-0.1	0.038
UBE2J1	0.13	0.0076
UQCC1	-0.12	0.013
VCAM1	0.19	0.00012
WASF2	-0.11	0.031
WDR1	0.1	0.034
XPR1	0.13	0.0063
ZBTB7A	-0.15	0.0027
ZBTB7B	-0.12	0.017
