# Supplementary material for: Prognostic Signature Development on the Basis of Macrophage Phagocytosis-Mediated Oxidative Phosphorylation in Bladder Cancer
Source: Oxid Med Cell Longev. 2022 Sep 29;2022:4754935. doi: 10.1155/2022/4754935 (PMC9537622; doi:10.1155/2022/4754935)
Supplement: Supplementary 8 — Supplementary Table 4: functional enrichment analysis of the MPOP: the list of GO enrichment analysis of MPOP. [file 4754935.f8.pdf]

	ONTOLOGY	ID	Description	GeneRatio	BgRatio	pvalue	p.adjust	qvalue	Count
GO:0033108	BP	GO:0033108	mitochondrial respiratory chain complex assembly	19/207	93/18723	4.10E-19	1.40E-15	1.08E-15	19
GO:0010257	BP	GO:0010257	NADH dehydrogenase complex assembly	15/207	57/18723	3.96E-17	4.51E-14	3.49E-14	15
GO:0032981	BP	GO:0032981	mitochondrial respiratory chain complex I assembly	15/207	57/18723	3.96E-17	4.51E-14	3.49E-14	15
GO:0006120	BP	GO:0006120	mitochondrial electron transport, NADH to ubiquinone	12/207	51/18723	2.63E-13	2.25E-10	1.74E-10	12
GO:0019646	BP	GO:0019646	aerobic electron transport chain	13/207	87/1872	1.26E-11	8.60E-09	6.66E-09	13
GO:0042773	BP	GO:0042773	ATP synthesis coupled electron transport	13/207	95/18723	3.98E-11	1.94E-08	1.50E-08	13
GO:0042775	BP	GO:0042775	mitochondrial ATP synthesis coupled electron transport	13/207	95/18723	3.98E-11	1.94E-08	1.50E-08	13
GO:0045785	BP	GO:0045785	positive regulation of cell adhesion	24/207	437/187	1.11E-10	4.73E-08	3.66E-08	24
GO:0050900	BP	GO:0050900	leukocyte migration	22/207	369/187	1.47E-10	5.57E-08	4.31E-08	22
GO:0007159	BP	GO:0007159	leukocyte cell-cell adhesion	22/207	371/187	1.63E-10	5.57E-08	4.31E-08	22
GO:0022904	BP	GO:0022904	respiratory electron transport chain	13/207	114/187	4.11E-10	1.17E-07	9.08E-08	13
GO:0022409	BP	GO:0022409	positive regulation of cell-cell adhesion	19/207	284/187	4.12E-10	1.17E-07	9.08E-08	19
GO:0006119	BP	GO:0006119	oxidative phosphorylation	14/207	141/187	5.53E-10	1.45E-07	1.12E-07	14
GO:0010592	BP	GO:0010592	positive regulation of lamellipodium assembly	8/207	29/18723	6.86E-10	1.67E-07	1.29E-07	8
GO:1903039	BP	GO:1903039	positive regulation of leukocyte cell-cell adhesion	17/207	239/18723	1.41E-09	3.20E-07	2.48E-07	17
GO:0009060	BP	GO:0009060	aerobic respiration	15/207	189/187	3.04E-09	6.48E-07	5.02E-07	15
GO:1902745	BP	GO:1902745	positive regulation of lamellipodium organization	8/207	37/18723	5.72E-09	1.10E-06	8.48E-07	8
GO:0030183	BP	GO:0030183	B cell differentiation	13/207	141/187	5.78E-09	1.10E-06	8.48E-07	13
GO:0030098	BP	GO:0030098	lymphocyte differentiation	20/207	374/187	6.81E-09	1.22E-06	9.48E-07	20

GO:1902743	BP	GO:1902743	regulation of lamellipodium organization	9/207	54/18723	7.17E-09	1.22E-06	9.48E-07	9
GO:0034314	BP	GO:0034314	Arp2/3 complex-mediated actin nucleation	8/207	39/18723	8.94E-09	1.45E-06	1.12E-06	8
GO:0022900	BP	GO:0022900	electron transport chain	14/207	175/187	9.35E-09	1.45E-06	1.12E-06	14
GO:1903131	BP	GO:1903131	mononuclear cell differentiation	21/207	426/187	1.17E-08	1.74E-06	1.35E-06	21
GO:0010591	BP	GO:0010591	regulation of lamellipodium assembly	8/207	42/18723	1.67E-08	2.37E-06	1.84E-06	8
GO:0008154	BP	GO:0008154	actin polymerization or depolymerization	15/207	218/187	2.11E-08	2.88E-06	2.23E-06	15
GO:0042110	BP	GO:0042110	T cell activation	22/207	487/187	2.48E-08	3.25E-06	2.52E-06	22
GO:0022407	BP	GO:0022407	regulation of cell-cell adhesion	21/207	448/187	2.80E-08	3.53E-06	2.74E-06	21
GO:0045333	BP	GO:0045333	cellular respiration	15/207	230/187	4.31E-08	5.25E-06	4.06E-06	15
GO:0032535	BP	GO:0032535	regulation of cellular component size	19/207	383/187	5.47E-08	6.43E-06	4.98E-06	19
GO:0097581	BP	GO:0097581	lamellipodium organization	10/207	90/1872	5.82E-08	6.62E-06	5.13E-06	10
GO:0048872	BP	GO:0048872	homeostasis of number of cells	16/207	272/187	6.36E-08	7.01E-06	5.43E-06	16
GO:0045010	BP	GO:0045010	actin nucleation	8/207	52/1872	9.67E-08	1.01E-05	7.81E-06	8
GO:0015980	BP	GO:0015980	energy derivation by oxidation of organic compounds	17/207	318/18723	9.75E-08	1.01E-05	7.81E-06	17
GO:0050870	BP	GO:0050870	positive regulation of T cell activation	14/207	216/187	1.33E-07	1.33E-05	1.03E-05	14
GO:0030838	BP	GO:0030838	positive regulation of actin filament polymerization	10/207	99/18723	1.45E-07	1.42E-05	1.10E-05	10
GO:0120032	BP	GO:0120032	regulation of plasma membrane bounded cell projection assembly	13/207	186/18723	1.58E-07	1.50E-05	1.16E-05	13
GO:0008064	BP	GO:0008064	regulation of actin polymerization or depolymerization	13/207	188/18723	1.79E-07	1.60E-05	1.24E-05	13
GO:0060491	BP	GO:0060491	regulation of cell projection assembly	13/207	188/187	1.79E-07	1.60E-05	1.24E-05	13
GO:0030832	BP	GO:0030832	regulation of actin filament length	13/207	189/187	1.90E-07	1.66E-05	1.29E-05	13
GO:1903037	BP	GO:1903037	regulation of leukocyte cell-cell adhesion	17/207	336/18723	2.14E-07	1.79E-05	1.38E-05	17

GO:0030041	BP	GO:0030041	actin filament polymerization	13/207	191/187	2.15E-07	1.79E-05	1.38E-05	13
GO:0002703	BP	GO:0002703	regulation of leukocyte mediated immunity	14/207	226/18723	2.32E-07	1.88E-05	1.46E-05	14
GO:0002687	BP	GO:0002687	positive regulation of leukocyte migration	11/207	135/187	3.13E-07	2.48E-05	1.92E-05	11
GO:0022604	BP	GO:0022604	regulation of cell morphogenesis	16/207	309/187	3.62E-07	2.80E-05	2.17E-05	16
GO:0032273	BP	GO:0032273	positive regulation of protein polymerization	11/207	138/18723	3.91E-07	2.96E-05	2.30E-05	11
GO:0046034	BP	GO:0046034	ATP metabolic process	15/207	277/187	4.82E-07	3.54E-05	2.74E-05	15
GO:0030833	BP	GO:0030833	regulation of actin filament polymerization	12/207	172/18723	4.88E-07	3.54E-05	2.74E-05	12
GO:0110053	BP	GO:0110053	regulation of actin filament organization	15/207	278/187	5.04E-07	3.58E-05	2.78E-05	15
GO:0006091	BP	GO:0006091	generation of precursor metabolites and energy	20/207	490/18723	5.58E-07	3.89E-05	3.01E-05	20
GO:1902905	BP	GO:1902905	positive regulation of supramolecular fiber organization	13/207	209/18723	6.03E-07	4.12E-05	3.19E-05	13
GO:1903706	BP	GO:1903706	regulation of hemopoiesis	17/207	367/187	7.35E-07	4.92E-05	3.81E-05	17
GO:0050863	BP	GO:0050863	regulation of T cell activation	16/207	329/187	8.33E-07	5.46E-05	4.23E-05	16
GO:0097529	BP	GO:0097529	myeloid leukocyte migration	13/207	220/187	1.08E-06	6.94E-05	5.37E-05	13
GO:0008360	BP	GO:0008360	regulation of cell shape	11/207	154/187	1.17E-06	7.39E-05	5.73E-05	11
GO:0002697	BP	GO:0002697	regulation of immune effector process	16/207	339/187	1.23E-06	7.62E-05	5.90E-05	16
GO:0030032	BP	GO:0030032	lamellipodium assembly	8/207	72/1872	1.27E-06	7.62E-05	5.90E-05	8
GO:0033627	BP	GO:0033627	cell adhesion mediated by integrin	8/207	72/1872	1.27E-06	7.62E-05	5.90E-05	8
GO:0043254	BP	GO:0043254	regulation of protein-containing complex assembly	18/207	428/18723	1.38E-06	8.13E-05	6.30E-05	18
GO:0051495	BP	GO:0051495	positive regulation of cytoskeleton organization	13/207	226/18723	1.46E-06	8.43E-05	6.53E-05	13
GO:0071674	BP	GO:0071674	mononuclear cell migration	12/207	196/187	1.95E-06	0.00011	8.55E-05	12
GO:0060326	BP	GO:0060326	cell chemotaxis	15/207	310/187	1.97E-06	0.00011	8.55E-05	15
GO:0032271	BP	GO:0032271	regulation of protein polymerization	13/207	233/187	2.05E-06	0.000113	8.72E-05	13

GO:0007015	BP	GO:0007015	actin filament organization	18/207	442/187	2.18E-06	0.000118	9.14E-05	18
GO:0031334	BP	GO:0031334	positive regulation of protein-containing complex assembly	13/207	237/18723	2.47E-06	0.000129	0.0001	13
GO:0120034	BP	GO:0120034	positive regulation of plasma membrane bounded cell projection assembly	9/207	105/18723	2.49E-06	0.000129	0.0001	9
GO:0032956	BP	GO:0032956	regulation of actin cytoskeleton organization	16/207	358/18723	2.50E-06	0.000129	0.0001	16
GO:0070661	BP	GO:0070661	leukocyte proliferation	15/207	318/187	2.70E-06	0.000138	0.000107	15
GO:0070663	BP	GO:0070663	regulation of leukocyte proliferation	13/207	245/18723	3.56E-06	0.000179	0.000138	13
GO:0045619	BP	GO:0045619	regulation of lymphocyte differentiation	11/207	174/18723	3.86E-06	0.000191	0.000148	11
GO:0002685	BP	GO:0002685	regulation of leukocyte migration	12/207	210/187	3.99E-06	0.000194	0.000151	12
GO:0046651	BP	GO:0046651	lymphocyte proliferation	14/207	288/187	4.14E-06	0.000199	0.000154	14
GO:0070371	BP	GO:0070371	ERK1 and ERK2 cascade	15/207	330/187	4.24E-06	0.000201	0.000156	15
GO:0032943	BP	GO:0032943	mononuclear cell proliferation	14/207	291/187	4.67E-06	0.000218	0.000169	14
GO:0030865	BP	GO:0030865	cortical cytoskeleton organization	7/207	61/1872	4.80E-06	0.000218	0.000169	7
GO:0034113	BP	GO:0034113	heterotypic cell-cell adhesion	7/207	61/1872	4.80E-06	0.000218	0.000169	7
GO:0042113	BP	GO:0042113	B cell activation	15/207	334/187	4.91E-06	0.00022	0.000171	15
GO:0032640	BP	GO:0032640	tumor necrosis factor production	11/207	181/187	5.64E-06	0.000247	0.000191	11
GO:0032680	BP	GO:0032680	regulation of tumor necrosis factor production	11/207	181/18723	5.64E-06	0.000247	0.000191	11
GO:0051258	BP	GO:0051258	protein polymerization	14/207	297/187	5.90E-06	0.000255	0.000197	14
GO:0071706	BP	GO:0071706	tumor necrosis factor superfamily cytokine production	11/207	186/18723	7.32E-06	0.000308	0.000239	11
GO:1903555	BP	GO:1903555	regulation of tumor necrosis factor superfamily cytokine production	11/207	186/18723	7.32E-06	0.000308	0.000239	11
GO:0032868	BP	GO:0032868	response to insulin	13/207	264/187	8.01E-06	0.000332	0.000257	13

GO:0050670	BP	GO:0050670	regulation of lymphocyte proliferation	12/207	225/18723	8.07E-06	0.000332	0.000257	12
GO:0032944	BP	GO:0032944	regulation of mononuclear cell proliferation	12/207	227/18723	8.83E-06	0.000359	0.000278	12
GO:0006909	BP	GO:0006909	phagocytosis	14/207	308/187	8.93E-06	0.000359	0.000278	14
GO:0032970	BP	GO:0032970	regulation of actin filament-based process	16/207	397/18723	9.29E-06	0.000369	0.000285	16
GO:0002690	BP	GO:0002690	positive regulation of leukocyte chemotaxis	8/207	94/1872	9.62E-06	0.000377	0.000292	8
GO:0046637	BP	GO:0046637	regulation of alpha-beta T cell differentiation	7/207	68/18723	9.99E-06	0.000387	0.0003	7
GO:0045123	BP	GO:0045123	cellular extravasation	7/207	70/1872	1.21E-05	0.000465	0.00036	7
GO:0002366	BP	GO:0002366	leukocyte activation involved in immune response	13/207	275/18723	1.24E-05	0.00047	0.000364	13
GO:0051251	BP	GO:0051251	positive regulation of lymphocyte activation	15/207	362/187	1.28E-05	0.000482	0.000373	15
GO:0061515	BP	GO:0061515	myeloid cell development	7/207	71/1872	1.33E-05	0.000493	0.000382	7
GO:0002696	BP	GO:0002696	positive regulation of leukocyte activation	16/207	409/187	1.34E-05	0.000493	0.000382	16
GO:0042098	BP	GO:0042098	T cell proliferation	11/207	199/187	1.39E-05	0.000503	0.00039	11
GO:0031341	BP	GO:0031341	regulation of cell killing	8/207	99/1872	1.41E-05	0.000503	0.00039	8
GO:0033628	BP	GO:0033628	regulation of cell adhesion mediated by integrin	6/207	48/18723	1.42E-05	0.000503	0.00039	6
GO:0002263	BP	GO:0002263	cell activation involved in immune response	13/207	279/18723	1.45E-05	0.000504	0.00039	13
GO:1902105	BP	GO:1902105	regulation of leukocyte differentiation	13/207	279/18723	1.45E-05	0.000504	0.00039	13
GO:0070085	BP	GO:0070085	glycosylation	12/207	240/187	1.54E-05	0.000533	0.000412	12
GO:0032869	BP	GO:0032869	cellular response to insulin stimulus	11/207	203/187	1.67E-05	0.00057	0.000441	11
GO:0050867	BP	GO:0050867	positive regulation of cell activation	16/207	420/187	1.86E-05	0.000629	0.000487	16
GO:0030148	BP	GO:0030148	sphingolipid biosynthetic process	8/207	103/187	1.89E-05	0.000631	0.000489	8
GO:0046634	BP	GO:0046634	regulation of alpha-beta T cell activation	8/207	104/187	2.02E-05	0.000671	0.00052	8
GO:0042129	BP	GO:0042129	regulation of T cell proliferation	10/207	171/187	2.11E-05	0.000693	0.000537	10

GO:0030099	BP	GO:0030099	myeloid cell differentiation	15/207	381/187	2.34E-05	0.000759	0.000588	15
GO:1902903	BP	GO:1902903	regulation of supramolecular fiber organization	15/207	383/18723	2.48E-05	0.000799	0.000619	15
GO:0046467	BP	GO:0046467	membrane lipid biosynthetic process	9/207	142/187	2.91E-05	0.000929	0.00072	9
GO:0035855	BP	GO:0035855	megakaryocyte development	4/207	17/1872	3.09E-05	0.000975	0.000755	4
GO:0002443	BP	GO:0002443	leukocyte mediated immunity	16/207	440/187	3.27E-05	0.001024	0.000793	16
GO:0002446	BP	GO:0002446	neutrophil mediated immunity	5/207	34/1872	3.37E-05	0.001047	0.000811	5
GO:0007162	BP	GO:0007162	negative regulation of cell adhesion	13/207	303/187	3.43E-05	0.001048	0.000811	13
GO:0001910	BP	GO:0001910	regulation of leukocyte mediated cytotoxicity	7/207	82/18723	3.44E-05	0.001048	0.000811	7
GO:0030219	BP	GO:0030219	megakaryocyte differentiation	6/207	57/1872	3.85E-05	0.001164	0.000901	6
GO:0097530	BP	GO:0097530	granulocyte migration	9/207	148/187	4.04E-05	0.001209	0.000936	9
GO:0071675	BP	GO:0071675	regulation of mononuclear cell migration	8/207	115/18723	4.19E-05	0.001236	0.000957	8
GO:0070372	BP	GO:0070372	regulation of ERK1 and ERK2 cascade	13/207	309/187	4.20E-05	0.001236	0.000957	13
GO:0008286	BP	GO:0008286	insulin receptor signaling pathway	8/207	116/187	4.46E-05	0.001266	0.000981	8
GO:0006486	BP	GO:0006486	protein glycosylation	11/207	226/187	4.49E-05	0.001266	0.000981	11
GO:0043413	BP	GO:0043413	macromolecule glycosylation	11/207	226/187	4.49E-05	0.001266	0.000981	11
GO:0070665	BP	GO:0070665	positive regulation of leukocyte proliferation	9/207	150/18723	4.49E-05	0.001266	0.000981	9
GO:0042119	BP	GO:0042119	neutrophil activation	5/207	36/1872	4.49E-05	0.001266	0.000981	5
GO:0001776	BP	GO:0001776	leukocyte homeostasis	7/207	87/1872	5.04E-05	0.001411	0.001093	7
GO:0030595	BP	GO:0030595	leukocyte chemotaxis	11/207	230/187	5.26E-05	0.001459	0.00113	11
GO:0032872	BP	GO:0032872	regulation of stress-activated MAPK cascade	10/207	192/18723	5.67E-05	0.00156	0.001208	10
GO:0032930	BP	GO:0032930	positive regulation of superoxide anion generation	4/207	20/18723	6.12E-05	0.001655	0.001281	4
GO:0002285	BP	GO:0002285	lymphocyte activation involved in immune response	10/207	194/18723	6.18E-05	0.001655	0.001281	10

GO:0016032	BP	GO:0016032	viral process	15/207	415/187	6.20E-05	0.001655	0.001281	15
GO:0002688	BP	GO:0002688	regulation of leukocyte chemotaxis	8/207	122/187	6.39E-05	0.001655	0.001281	8
GO:1990266	BP	GO:1990266	neutrophil migration	8/207	122/187	6.39E-05	0.001655	0.001281	8
GO:0002262	BP	GO:0002262	myeloid cell homeostasis	9/207	157/187	6.40E-05	0.001655	0.001281	9
GO:1902107	BP	GO:1902107	positive regulation of leukocyte differentiation	9/207	157/18723	6.40E-05	0.001655	0.001281	9
GO:1903708	BP	GO:1903708	positive regulation of hemopoiesis	9/207	157/187	6.40E-05	0.001655	0.001281	9
GO:0070302	BP	GO:0070302	regulation of stress-activated protein kinase signaling cascade	10/207	195/18723	6.46E-05	0.001657	0.001283	10
GO:0051051	BP	GO:0051051	negative regulation of transport	16/207	470/187	7.16E-05	0.001812	0.001404	16
GO:0001909	BP	GO:0001909	leukocyte mediated cytotoxicity	8/207	124/187	7.17E-05	0.001812	0.001404	8
GO:0033630	BP	GO:0033630	positive regulation of cell adhesion mediated by integrin	4/207	21/18723	7.49E-05	0.001881	0.001457	4
GO:0071621	BP	GO:0071621	granulocyte chemotaxis	8/207	125/187	7.59E-05	0.00189	0.001464	8
GO:0006487	BP	GO:0006487	protein N-linked glycosylation	6/207	65/1872	8.15E-05	0.002002	0.00155	6
GO:0046513	BP	GO:0046513	ceramide biosynthetic process	6/207	65/1872	8.15E-05	0.002002	0.00155	6
GO:0050764	BP	GO:0050764	regulation of phagocytosis	7/207	95/1872	8.86E-05	0.002159	0.001672	7
GO:0034101	BP	GO:0034101	erythrocyte homeostasis	8/207	129/187	9.47E-05	0.002292	0.001775	8
GO:0016601	BP	GO:0016601	Rac protein signal transduction	5/207	42/1872	9.60E-05	0.002307	0.001787	5
GO:0002429	BP	GO:0002429	immune response-activating cell surface receptor signaling pathway	12/207	291/18723	0.0001	0.002379	0.001842	12
GO:0002757	BP	GO:0002757	immune response-activating signal transduction	12/207	291/18723	0.0001	0.002379	0.001842	12
GO:0001655	BP	GO:0001655	urogenital system development	13/207	338/187	0.000104	0.002453	0.0019	13
GO:0016266	BP	GO:0016266	O-glycan processing	5/207	43/1872	0.000108	0.002486	0.001925	5
GO:0036230	BP	GO:0036230	granulocyte activation	5/207	43/1872	0.000108	0.002486	0.001925	5
GO:0070301	BP	GO:0070301	cellular response to hydrogen peroxide	7/207	98/1872	0.000108	0.002486	0.001925	7

GO:0050765	BP	GO:0050765	negative regulation of phagocytosis	4/207	23/1872	0.000109	0.002496	0.001933	4
GO:0007219	BP	GO:0007219	Notch signaling pathway	9/207	172/187	0.000129	0.002889	0.002237	9
GO:0032528	BP	GO:0032528	microvillus organization	4/207	24/1872	0.00013	0.002889	0.002237	4
GO:0032928	BP	GO:0032928	regulation of superoxide anion generation	4/207	24/18723	0.00013	0.002889	0.002237	4
GO:0090023	BP	GO:0090023	positive regulation of neutrophil chemotaxis	4/207	24/1872	0.00013	0.002889	0.002237	4
GO:0042102	BP	GO:0042102	positive regulation of T cell proliferation	7/207	101/187	0.00013	0.002889	0.002237	7
GO:0030217	BP	GO:0030217	T cell differentiation	11/207	257/187	0.000141	0.003111	0.002409	11
GO:0050671	BP	GO:0050671	positive regulation of lymphocyte proliferation	8/207	137/18723	0.000144	0.003151	0.00244	8
GO:0030593	BP	GO:0030593	neutrophil chemotaxis	7/207	103/187	0.000147	0.003204	0.002481	7
GO:0007157	BP	GO:0007157	heterophilic cell-cell adhesion via plasma membrane cell adhesion molecules	5/207	46/18723	0.000149	0.003204	0.002481	5
GO:0043300	BP	GO:0043300	regulation of leukocyte degranulation	5/207	46/18723	0.000149	0.003204	0.002481	5
GO:2000601	BP	GO:2000601	positive regulation of Arp2/3 complex-mediated actin nucleation	3/207	10/18723	0.000151	0.003211	0.002487	3
GO:0032946	BP	GO:0032946	positive regulation of mononuclear cell proliferation	8/207	138/18723	0.000151	0.003211	0.002487	8
GO:0045621	BP	GO:0045621	positive regulation of lymphocyte differentiation	7/207	104/18723	0.000157	0.003299	0.002555	7
GO:0007163	BP	GO:0007163	establishment or maintenance of cell polarity	10/207	218/18723	0.000162	0.003395	0.00263	10
GO:0032008	BP	GO:0032008	positive regulation of TOR signaling	5/207	47/1872	0.000166	0.003445	0.002668	5
GO:0034341	BP	GO:0034341	response to interferon-gamma	8/207	141/187	0.000176	0.003612	0.002798	8
GO:0050921	BP	GO:0050921	positive regulation of chemotaxis	8/207	141/187	0.000176	0.003612	0.002798	8
GO:0043409	BP	GO:0043409	negative regulation of MAPK cascade	9/207	180/187	0.000182	0.003711	0.002874	9
GO:1901653	BP	GO:1901653	cellular response to peptide	13/207	359/187	0.000189	0.003839	0.002973	13

GO:0002274	BP	GO:0002274	myeloid leukocyte activation	10/207	223/187	0.000195	0.003936	0.003048	10
GO:0006054	BP	GO:0006054	N-acetylneuraminate metabolic process	3/207	11/1872	0.000206	0.004059	0.003144	3
GO:0043312	BP	GO:0043312	neutrophil degranulation	3/207	11/1872	0.000206	0.004059	0.003144	3
GO:0046643	BP	GO:0046643	regulation of gamma-delta T cell activation	3/207	11/18723	0.000206	0.004059	0.003144	3
GO:0002433	BP	GO:0002433	immune response-regulating cell surface receptor signaling pathway involved in phagocytosis	4/207	27/18723	0.000209	0.004059	0.003144	4
GO:0038096	BP	GO:0038096	Fc-gamma receptor signaling pathway involved in phagocytosis	4/207	27/18723	0.000209	0.004059	0.003144	4
GO:0071624	BP	GO:0071624	positive regulation of granulocyte chemotaxis	4/207	27/18723	0.000209	0.004059	0.003144	4
GO:0002768	BP	GO:0002768	immune response-regulating cell surface receptor signaling pathway	12/207	315/18723	0.00021	0.004059	0.003144	12
GO:0031589	BP	GO:0031589	cell-substrate adhesion	13/207	363/187	0.000211	0.004059	0.003144	13
GO:0002698	BP	GO:0002698	negative regulation of immune effector process	7/207	110/18723	0.000222	0.004207	0.003258	7
GO:0009101	BP	GO:0009101	glycoprotein biosynthetic process	12/207	317/187	0.000223	0.004207	0.003258	12
GO:0060337	BP	GO:0060337	type I interferon signaling pathway	5/207	50/1872	0.000223	0.004207	0.003258	5
GO:0045580	BP	GO:0045580	regulation of T cell differentiation	8/207	146/187	0.000223	0.004207	0.003258	8
GO:0038094	BP	GO:0038094	Fc-gamma receptor signaling pathway	4/207	28/1872	0.000241	0.004488	0.003475	4
GO:1902624	BP	GO:1902624	positive regulation of neutrophil migration	4/207	28/1872	0.000241	0.004488	0.003475	4
GO:0071496	BP	GO:0071496	cellular response to external stimulus	12/207	320/187	0.000243	0.004488	0.003475	12
GO:0043370	BP	GO:0043370	regulation of CD4-positive, alpha-beta T cell differentiation	5/207	51/18723	0.000245	0.004488	0.003475	5
GO:0071622	BP	GO:0071622	regulation of granulocyte chemotaxis	5/207	51/18723	0.000245	0.004488	0.003475	5
GO:0046632	BP	GO:0046632	alpha-beta T cell differentiation	7/207	112/187	0.000248	0.004525	0.003504	7

GO:0001906	BP	GO:0001906	cell killing	9/207	188/187	0.000251	0.004559	0.003531	9
GO:0071357	BP	GO:0071357	cellular response to type I interferon	5/207	52/1872	0.000268	0.004843	0.00375	5
GO:0072683	BP	GO:0072683	T cell extravasation	3/207	12/1872	0.000272	0.004865	0.003767	3
GO:1902563	BP	GO:1902563	regulation of neutrophil activation	3/207	12/1872	0.000272	0.004865	0.003767	3
GO:0002253	BP	GO:0002253	activation of immune response	13/207	375/187	0.000288	0.005124	0.003968	13
GO:0008625	BP	GO:0008625	extrinsic apoptotic signaling pathway via death domain receptors	6/207	82/18723	0.000296	0.005231	0.004051	6
GO:0032103	BP	GO:0032103	positive regulation of response to external stimulus	14/207	427/18723	0.000298	0.005249	0.004065	14
GO:0045577	BP	GO:0045577	regulation of B cell differentiation	4/207	30/1872	0.000317	0.005557	0.004303	4
GO:0002823	BP	GO:0002823	negative regulation of adaptive immune response based on somatic recombination of immune receptors built from immunoglobulin superfamily domains	5/207	54/18723	0.000321	0.005582	0.004323	5
GO:0006665	BP	GO:0006665	sphingolipid metabolic process	8/207	155/187	0.000335	0.00577	0.004468	8
GO:0034614	BP	GO:0034614	cellular response to reactive oxygen species	8/207	155/18723	0.000335	0.00577	0.004468	8
GO:0051403	BP	GO:0051403	stress-activated MAPK cascade	10/207	239/187	0.000339	0.00582	0.004507	10
GO:0071346	BP	GO:0071346	cellular response to interferon-gamma	7/207	118/187	0.000341	0.005824	0.00451	7
GO:0046631	BP	GO:0046631	alpha-beta T cell activation	8/207	156/187	0.00035	0.005873	0.004548	8
GO:0036462	BP	GO:0036462	TRAIL-activated apoptotic signaling pathway	3/207	13/18723	0.000351	0.005873	0.004548	3
GO:0042492	BP	GO:0042492	gamma-delta T cell differentiation	3/207	13/1872	0.000351	0.005873	0.004548	3
GO:0070486	BP	GO:0070486	leukocyte aggregation	3/207	13/1872	0.000351	0.005873	0.004548	3
GO:0030218	BP	GO:0030218	erythrocyte differentiation	7/207	120/187	0.000378	0.006293	0.004873	7
GO:0002886	BP	GO:0002886	regulation of myeloid leukocyte mediated immunity	5/207	56/18723	0.00038	0.006301	0.00488	5

GO:0071375	BP	GO:0071375	cellular response to peptide hormone stimulus	11/207	290/18723	0.000399	0.006578	0.005095	11
GO:0090022	BP	GO:0090022	regulation of neutrophil chemotaxis	4/207	32/1872	0.000409	0.006718	0.005203	4
GO:0031098	BP	GO:0031098	stress-activated protein kinase signaling cascade	10/207	247/18723	0.00044	0.007168	0.005551	10
GO:0006643	BP	GO:0006643	membrane lipid metabolic process	9/207	203/187	0.000441	0.007168	0.005551	9
GO:0003376	BP	GO:0003376	sphingosine-1-phosphate receptor signaling pathway	3/207	14/18723	0.000443	0.007168	0.005551	3
GO:0034340	BP	GO:0034340	response to type I interferon	5/207	58/1872	0.000448	0.007215	0.005587	5
GO:0032496	BP	GO:0032496	response to lipopolysaccharide	12/207	343/187	0.000454	0.007272	0.005632	12
GO:0002431	BP	GO:0002431	Fc receptor mediated stimulatory signaling pathway	4/207	33/18723	0.000462	0.007367	0.005705	4
GO:0060348	BP	GO:0060348	bone development	9/207	205/187	0.000474	0.007522	0.005825	9
GO:0002820	BP	GO:0002820	negative regulation of adaptive immune response	5/207	59/18723	0.000485	0.007668	0.005938	5
GO:2001235	BP	GO:2001235	positive regulation of apoptotic signaling pathway	7/207	126/18723	0.000507	0.007981	0.006181	7
GO:0045582	BP	GO:0045582	positive regulation of T cell differentiation	6/207	91/1872	0.000519	0.008088	0.006264	6
GO:0098751	BP	GO:0098751	bone cell development	4/207	34/1872	0.000519	0.008088	0.006264	4
GO:0051127	BP	GO:0051127	positive regulation of actin nucleation	3/207	15/1872	0.000549	0.008485	0.006571	3
GO:0072075	BP	GO:0072075	metanephric mesenchyme development	3/207	15/18723	0.000549	0.008485	0.006571	3
GO:0072001	BP	GO:0072001	renal system development	11/207	302/187	0.00056	0.00861	0.006668	11
GO:0050866	BP	GO:0050866	negative regulation of cell activation	9/207	210/187	0.000564	0.00863	0.006683	9
GO:0002706	BP	GO:0002706	regulation of lymphocyte mediated immunity	8/207	168/18723	0.000573	0.008687	0.006727	8
GO:0002822	BP	GO:0002822	regulation of adaptive immune response based on somatic recombination of immune receptors built from immunoglobulin superfamily domains	8/207	168/18723	0.000573	0.008687	0.006727	8

GO:2001233	BP	GO:2001233	regulation of apoptotic signaling pathway	12/207	356/18723	0.000631	0.009523	0.007375	12
GO:0045089	BP	GO:0045089	positive regulation of innate immune response	7/207	131/18723	0.00064	0.009578	0.007417	7
GO:0050853	BP	GO:0050853	B cell receptor signaling pathway	7/207	131/187	0.00064	0.009578	0.007417	7
GO:0060338	BP	GO:0060338	regulation of type I interferon-mediated signaling pathway	4/207	36/18723	0.000648	0.009578	0.007417	4
GO:0090322	BP	GO:0090322	regulation of superoxide metabolic process	4/207	36/18723	0.000648	0.009578	0.007417	4
GO:0032479	BP	GO:0032479	regulation of type I interferon production	6/207	95/1872	0.000652	0.009578	0.007417	6
GO:0032606	BP	GO:0032606	type I interferon production	6/207	95/1872	0.000652	0.009578	0.007417	6
GO:0002704	BP	GO:0002704	negative regulation of leukocyte mediated immunity	5/207	63/18723	0.000657	0.009578	0.007417	5
GO:0031343	BP	GO:0031343	positive regulation of cell killing	5/207	63/1872	0.000657	0.009578	0.007417	5
GO:0007569	BP	GO:0007569	cell aging	7/207	132/187	0.00067	0.009578	0.007417	7
GO:0002693	BP	GO:0002693	positive regulation of cellular extravasation	3/207	16/1872	0.000671	0.009578	0.007417	3
GO:0030033	BP	GO:0030033	microvillus assembly	3/207	16/1872	0.000671	0.009578	0.007417	3
GO:0071732	BP	GO:0071732	cellular response to nitric oxide	3/207	16/1872	0.000671	0.009578	0.007417	3
GO:0090520	BP	GO:0090520	sphingolipid mediated signaling pathway	3/207	16/18723	0.000671	0.009578	0.007417	3
GO:0030888	BP	GO:0030888	regulation of B cell proliferation	5/207	64/1872	0.000707	0.01001	0.007752	5
GO:1900076	BP	GO:1900076	regulation of cellular response to insulin stimulus	5/207	64/18723	0.000707	0.01001	0.007752	5
GO:0043434	BP	GO:0043434	response to peptide hormone	13/207	414/187	0.000731	0.010316	0.007989	13
GO:0045088	BP	GO:0045088	regulation of innate immune response	9/207	218/187	0.000737	0.01034	0.008008	9
GO:0002764	BP	GO:0002764	immune response-regulating signaling pathway	14/207	468/18723	0.000739	0.01034	0.008008	14
GO:0002237	BP	GO:0002237	response to molecule of bacterial origin	12/207	363/187	0.000748	0.010415	0.008066	12
GO:0071677	BP	GO:0071677	positive regulation of mononuclear cell migration	5/207	65/18723	0.000759	0.01053	0.008154	5

GO:0035337	BP	GO:0035337	fatty-acyl-CoA metabolic process	4/207	38/1872	0.000798	0.010979	0.008503	4
GO:1903305	BP	GO:1903305	regulation of regulated secretory pathway	7/207	136/18723	0.0008	0.010979	0.008503	7
GO:0072673	BP	GO:0072673	lamellipodium morphogenesis	3/207	17/1872	0.000808	0.010979	0.008503	3
GO:0002444	BP	GO:0002444	myeloid leukocyte mediated immunity	6/207	99/1872	0.000811	0.010979	0.008503	6
GO:0042100	BP	GO:0042100	B cell proliferation	6/207	99/1872	0.000811	0.010979	0.008503	6
GO:0046626	BP	GO:0046626	regulation of insulin receptor signaling pathway	5/207	66/18723	0.000814	0.010979	0.008503	5
GO:0072678	BP	GO:0072678	T cell migration	5/207	66/1872	0.000814	0.010979	0.008503	5
GO:0019058	BP	GO:0019058	viral life cycle	11/207	317/187	0.000835	0.011193	0.008668	11
GO:0035265	BP	GO:0035265	organ growth	8/207	178/187	0.000836	0.011193	0.008668	8
GO:0046635	BP	GO:0046635	positive regulation of alpha-beta T cell activation	5/207	67/18723	0.000872	0.011533	0.008932	5
GO:2000514	BP	GO:2000514	regulation of CD4-positive, alpha-beta T cell activation	5/207	67/18723	0.000872	0.011533	0.008932	5
GO:0072594	BP	GO:0072594	establishment of protein localization to organelle	13/207	422/18723	0.000872	0.011533	0.008932	13
GO:0048608	BP	GO:0048608	reproductive structure development	13/207	424/18723	0.00091	0.011995	0.00929	13
GO:0043410	BP	GO:0043410	positive regulation of MAPK cascade	14/207	480/187	0.000943	0.012383	0.00959	14
GO:0006672	BP	GO:0006672	ceramide metabolic process	6/207	102/187	0.000948	0.012396	0.0096	6
GO:0002283	BP	GO:0002283	neutrophil activation involved in immune response	3/207	18/18723	0.000962	0.01243	0.009627	3
GO:1902170	BP	GO:1902170	cellular response to reactive nitrogen species	3/207	18/18723	0.000962	0.01243	0.009627	3
GO:2000641	BP	GO:2000641	regulation of early endosome to late endosome transport	3/207	18/18723	0.000962	0.01243	0.009627	3
GO:0061458	BP	GO:0061458	reproductive system development	13/207	427/187	0.000971	0.012461	0.009651	13
GO:0030866	BP	GO:0030866	cortical actin cytoskeleton organization	4/207	40/18723	0.000971	0.012461	0.009651	4
GO:0018108	BP	GO:0018108	peptidyl-tyrosine phosphorylation	12/207	375/187	0.000991	0.012662	0.009806	12

GO:0032760	BP	GO:0032760	positive regulation of tumor necrosis factor production	6/207	103/18723	0.000997	0.012694	0.009831	6
GO:0002819	BP	GO:0002819	regulation of adaptive immune response	8/207	183/187	0.001	0.012694	0.009831	8
GO:0032006	BP	GO:0032006	regulation of TOR signaling	6/207	104/187	0.001049	0.013259	0.010268	6
GO:0018212	BP	GO:0018212	peptidyl-tyrosine modification	12/207	378/187	0.001061	0.013357	0.010344	12
GO:1902622	BP	GO:1902622	regulation of neutrophil migration	4/207	41/1872	0.001067	0.013388	0.010368	4
GO:0002683	BP	GO:0002683	negative regulation of immune system process	13/207	434/18723	0.001124	0.013873	0.010744	13
GO:0014067	BP	GO:0014067	negative regulation of phosphatidylinositol 3-kinase signaling	3/207	19/18723	0.001133	0.013873	0.010744	3
GO:0030011	BP	GO:0030011	maintenance of cell polarity	3/207	19/1872	0.001133	0.013873	0.010744	3
GO:0030220	BP	GO:0030220	platelet formation	3/207	19/1872	0.001133	0.013873	0.010744	3
GO:0036344	BP	GO:0036344	platelet morphogenesis	3/207	19/1872	0.001133	0.013873	0.010744	3
GO:0072074	BP	GO:0072074	kidney mesenchyme development	3/207	19/1872	0.001133	0.013873	0.010744	3
GO:0150116	BP	GO:0150116	regulation of cell-substrate junction organization	5/207	71/18723	0.001134	0.013873	0.010744	5
GO:0002695	BP	GO:0002695	negative regulation of leukocyte activation	8/207	187/187	0.00115	0.014013	0.010852	8
GO:0046620	BP	GO:0046620	regulation of organ growth	6/207	106/187	0.001158	0.014065	0.010892	6
GO:0006040	BP	GO:0006040	amino sugar metabolic process	4/207	42/1872	0.001169	0.014101	0.01092	4
GO:0030890	BP	GO:0030890	positive regulation of B cell proliferation	4/207	42/1872	0.001169	0.014101	0.01092	4
GO:0007160	BP	GO:0007160	cell-matrix adhesion	9/207	233/187	0.001177	0.014144	0.010954	9
GO:0006470	BP	GO:0006470	protein dephosphorylation	10/207	281/187	0.001183	0.014169	0.010973	10
GO:0042542	BP	GO:0042542	response to hydrogen peroxide	7/207	146/187	0.001211	0.014406	0.011157	7
GO:0007229	BP	GO:0007229	integrin-mediated signaling pathway	6/207	107/187	0.001216	0.014406	0.011157	6
GO:1903557	BP	GO:1903557	positive regulation of tumor necrosis factor superfamily cytokine production	6/207	107/18723	0.001216	0.014406	0.011157	6
GO:0043299	BP	GO:0043299	leukocyte degranulation	5/207	73/1872	0.001285	0.015178	0.011755	5

GO:0009100	BP	GO:0009100	glycoprotein metabolic process	12/207	387/187	0.001296	0.015251	0.011811	12
GO:0032986	BP	GO:0032986	protein-DNA complex disassembly	3/207	20/1872	0.001322	0.015342	0.011882	3
GO:0046629	BP	GO:0046629	gamma-delta T cell activation	3/207	20/1872	0.001322	0.015342	0.011882	3
GO:0071731	BP	GO:0071731	response to nitric oxide	3/207	20/1872	0.001322	0.015342	0.011882	3
GO:0097503	BP	GO:0097503	sialylation	3/207	20/1872	0.001322	0.015342	0.011882	3
GO:0002456	BP	GO:0002456	T cell mediated immunity	6/207	109/187	0.001338	0.015476	0.011985	6
GO:0032386	BP	GO:0032386	regulation of intracellular transport	11/207	337/187	0.001366	0.015753	0.012199	11
GO:0042554	BP	GO:0042554	superoxide anion generation	4/207	44/1872	0.001394	0.016017	0.012404	4
GO:0001959	BP	GO:0001959	regulation of cytokine-mediated signaling pathway	7/207	150/18723	0.001416	0.016217	0.012559	7
GO:0097193	BP	GO:0097193	intrinsic apoptotic signaling pathway	10/207	288/187	0.001422	0.016229	0.012568	10
GO:0007568	BP	GO:0007568	aging	11/207	339/187	0.001432	0.016291	0.012616	11
GO:0050851	BP	GO:0050851	antigen receptor-mediated signaling pathway	9/207	240/18723	0.001445	0.016384	0.012688	9
GO:0034504	BP	GO:0034504	protein localization to nucleus	10/207	290/187	0.001497	0.016915	0.013099	10
GO:0034315	BP	GO:0034315	regulation of Arp2/3 complex-mediated actin nucleation	3/207	21/18723	0.001529	0.017208	0.013327	3
GO:0032609	BP	GO:0032609	interferon-gamma production	6/207	112/187	0.001538	0.017208	0.013327	6
GO:0032649	BP	GO:0032649	regulation of interferon-gamma production	6/207	112/18723	0.001538	0.017208	0.013327	6
GO:0022408	BP	GO:0022408	negative regulation of cell-cell adhesion	8/207	196/187	0.00155	0.017293	0.013392	8
GO:0001822	BP	GO:0001822	kidney development	10/207	293/187	0.001615	0.017955	0.013905	10
GO:2000107	BP	GO:2000107	negative regulation of leukocyte apoptotic process	4/207	46/18723	0.001647	0.01825	0.014134	4
GO:0002286	BP	GO:0002286	T cell activation involved in immune response	6/207	114/18723	0.001683	0.018594	0.0144	6
GO:0006606	BP	GO:0006606	protein import into nucleus	7/207	155/187	0.001709	0.018726	0.014502	7
GO:0031668	BP	GO:0031668	cellular response to extracellular stimulus	9/207	246/187	0.001712	0.018726	0.014502	9

GO:0071216	BP	GO:0071216	cellular response to biotic stimulus	9/207	246/187	0.001712	0.018726	0.014502	9
GO:0070373	BP	GO:0070373	negative regulation of ERK1 and ERK2 cascade	5/207	78/18723	0.001728	0.018842	0.014592	5
GO:0032069	BP	GO:0032069	regulation of nuclease activity	3/207	22/1872	0.001757	0.018971	0.014692	3
GO:0043371	BP	GO:0043371	negative regulation of CD4-positive, alpha-beta T cell differentiation	3/207	22/18723	0.001757	0.018971	0.014692	3
GO:0046628	BP	GO:0046628	positive regulation of insulin receptor signaling pathway	3/207	22/18723	0.001757	0.018971	0.014692	3
GO:0045646	BP	GO:0045646	regulation of erythrocyte differentiation	4/207	47/18723	0.001785	0.019216	0.014881	4
GO:0018107	BP	GO:0018107	peptidyl-threonine phosphorylation	6/207	116/187	0.001839	0.019678	0.01524	6
GO:0043200	BP	GO:0043200	response to amino acid	6/207	116/187	0.001839	0.019678	0.01524	6
GO:0032388	BP	GO:0032388	positive regulation of intracellular transport	8/207	202/187	0.001874	0.019989	0.01548	8
GO:0072676	BP	GO:0072676	lymphocyte migration	6/207	117/187	0.001921	0.020426	0.015819	6
GO:2001238	BP	GO:2001238	positive regulation of extrinsic apoptotic signaling pathway	4/207	48/18723	0.00193	0.020461	0.015846	4
GO:0051170	BP	GO:0051170	import into nucleus	7/207	159/187	0.001976	0.020875	0.016166	7
GO:0018279	BP	GO:0018279	protein N-linked glycosylation via asparagine	3/207	23/18723	0.002004	0.021041	0.016295	3
GO:1903306	BP	GO:1903306	negative regulation of regulated secretory pathway	3/207	23/18723	0.002004	0.021041	0.016295	3
GO:2000106	BP	GO:2000106	regulation of leukocyte apoptotic process	5/207	81/18723	0.002042	0.021378	0.016556	5
GO:0035088	BP	GO:0035088	establishment or maintenance of apical/basal cell polarity	4/207	49/18723	0.002084	0.021619	0.016743	4
GO:0061245	BP	GO:0061245	establishment or maintenance of bipolar cell polarity	4/207	49/18723	0.002084	0.021619	0.016743	4
GO:1902041	BP	GO:1902041	regulation of extrinsic apoptotic signaling pathway via death domain receptors	4/207	49/18723	0.002084	0.021619	0.016743	4
GO:0017038	BP	GO:0017038	protein import	8/207	206/187	0.002118	0.021906	0.016965	8

GO:0002312	BP	GO:0002312	B cell activation involved in immune response	5/207	82/18723	0.002155	0.022225	0.017212	5
GO:0060759	BP	GO:0060759	regulation of response to cytokine stimulus	7/207	162/18723	0.002196	0.022574	0.017482	7
GO:0038093	BP	GO:0038093	Fc receptor signaling pathway	4/207	50/1872	0.002246	0.022881	0.01772	4
GO:0046638	BP	GO:0046638	positive regulation of alpha-beta T cell differentiation	4/207	50/18723	0.002246	0.022881	0.01772	4
GO:1901570	BP	GO:1901570	fatty acid derivative biosynthetic process	4/207	50/1872	0.002246	0.022881	0.01772	4
GO:0001779	BP	GO:0001779	natural killer cell differentiation	3/207	24/1872	0.002271	0.022955	0.017777	3
GO:0018196	BP	GO:0018196	peptidyl-asparagine modification	3/207	24/1872	0.002271	0.022955	0.017777	3
GO:0043367	BP	GO:0043367	CD4-positive, alpha-beta T cell differentiation	5/207	83/18723	0.002273	0.022955	0.017777	5
GO:0071222	BP	GO:0071222	cellular response to lipopolysaccharide	8/207	209/187	0.002317	0.023326	0.018064	8
GO:0045637	BP	GO:0045637	regulation of myeloid cell differentiation	8/207	210/187	0.002386	0.023954	0.018551	8
GO:0032873	BP	GO:0032873	negative regulation of stress-activated MAPK cascade	4/207	51/18723	0.002416	0.024112	0.018674	4
GO:0070303	BP	GO:0070303	negative regulation of stress-activated protein kinase signaling cascade	4/207	51/18723	0.002416	0.024112	0.018674	4
GO:0032635	BP	GO:0032635	interleukin-6 production	7/207	165/187	0.002435	0.024155	0.018707	7
GO:0032675	BP	GO:0032675	regulation of interleukin-6 production	7/207	165/18723	0.002435	0.024155	0.018707	7
GO:0030168	BP	GO:0030168	platelet activation	6/207	123/187	0.002472	0.024384	0.018884	6
GO:0050852	BP	GO:0050852	T cell receptor signaling pathway	6/207	123/187	0.002472	0.024384	0.018884	6
GO:0002709	BP	GO:0002709	regulation of T cell mediated immunity	5/207	85/1872	0.002523	0.024817	0.019219	5
GO:0035336	BP	GO:0035336	long-chain fatty-acyl-CoA metabolic process	3/207	25/18723	0.00256	0.024966	0.019334	3
GO:0046949	BP	GO:0046949	fatty-acyl-CoA biosynthetic process	3/207	25/1872	0.00256	0.024966	0.019334	3
GO:1900078	BP	GO:1900078	positive regulation of cellular response to insulin stimulus	3/207	25/18723	0.00256	0.024966	0.019334	3
GO:0043277	BP	GO:0043277	apoptotic cell clearance	4/207	52/1872	0.002595	0.025235	0.019543	4

GO:0006493	BP	GO:0006493	protein O-linked glycosylation	5/207	86/1872	0.002655	0.025637	0.019855	5
GO:0022406	BP	GO:0022406	membrane docking	5/207	86/1872	0.002655	0.025637	0.019855	5
GO:0042060	BP	GO:0042060	wound healing	12/207	422/187	0.002659	0.025637	0.019855	12
GO:0018210	BP	GO:0018210	peptidyl-threonine modification	6/207	125/187	0.00268	0.025762	0.019951	6
GO:0002833	BP	GO:0002833	positive regulation of response to biotic stimulus	7/207	168/18723	0.002693	0.025818	0.019994	7
GO:0018105	BP	GO:0018105	peptidyl-serine phosphorylation	10/207	315/187	0.002735	0.026147	0.020249	10
GO:0050730	BP	GO:0050730	regulation of peptidyl-tyrosine phosphorylation	9/207	264/18723	0.002757	0.026284	0.020355	9
GO:0002707	BP	GO:0002707	negative regulation of lymphocyte mediated immunity	4/207	53/18723	0.002783	0.026434	0.020472	4
GO:0031929	BP	GO:0031929	TOR signaling	6/207	126/187	0.002788	0.026434	0.020472	6
GO:0046639	BP	GO:0046639	negative regulation of alpha-beta T cell differentiation	3/207	26/18723	0.002871	0.027141	0.021019	3
GO:0070374	BP	GO:0070374	positive regulation of ERK1 and ERK2 cascade	8/207	217/18723	0.002919	0.027506	0.021302	8
GO:0031331	BP	GO:0031331	positive regulation of cellular catabolic process	12/207	427/18723	0.002926	0.027506	0.021302	12
GO:0070228	BP	GO:0070228	regulation of lymphocyte apoptotic process	4/207	54/18723	0.00298	0.027942	0.02164	4
GO:2001020	BP	GO:2001020	regulation of response to DNA damage stimulus	8/207	219/18723	0.003087	0.028868	0.022357	8
GO:0034109	BP	GO:0034109	homotypic cell-cell adhesion	5/207	90/1872	0.003234	0.030077	0.023292	5
GO:0035304	BP	GO:0035304	regulation of protein dephosphorylation	5/207	90/1872	0.003234	0.030077	0.023292	5
GO:0010810	BP	GO:0010810	regulation of cell-substrate adhesion	8/207	221/187	0.003263	0.030179	0.023372	8
GO:0071219	BP	GO:0071219	cellular response to molecule of bacterial origin	8/207	221/18723	0.003263	0.030179	0.023372	8
GO:0000302	BP	GO:0000302	response to reactive oxygen species	8/207	222/187	0.003353	0.030933	0.023956	8
GO:0002275	BP	GO:0002275	myeloid cell activation involved in immune response	5/207	91/18723	0.003392	0.030965	0.023981	5
GO:0050878	BP	GO:0050878	regulation of body fluid levels	11/207	379/187	0.003397	0.030965	0.023981	11

GO:0001912	BP	GO:0001912	positive regulation of leukocyte mediated cytotoxicity	4/207	56/18723	0.003402	0.030965	0.023981	4
GO:0032608	BP	GO:0032608	interferon-beta production	4/207	56/1872	0.003402	0.030965	0.023981	4
GO:0032648	BP	GO:0032648	regulation of interferon-beta production	4/207	56/18723	0.003402	0.030965	0.023981	4
GO:0050920	BP	GO:0050920	regulation of chemotaxis	8/207	223/187	0.003446	0.03128	0.024225	8
GO:0002313	BP	GO:0002313	mature B cell differentiation involved in immune response	3/207	28/18723	0.003559	0.032044	0.024816	3
GO:0090344	BP	GO:0090344	negative regulation of cell aging	3/207	28/1872	0.003559	0.032044	0.024816	3
GO:1903649	BP	GO:1903649	regulation of cytoplasmic transport	3/207	28/1872	0.003559	0.032044	0.024816	3
GO:0002831	BP	GO:0002831	regulation of response to biotic stimulus	10/207	327/18723	0.003568	0.032044	0.024816	10
GO:0061756	BP	GO:0061756	leukocyte adhesion to vascular endothelial cell	4/207	57/18723	0.003628	0.032498	0.025168	4
GO:0090398	BP	GO:0090398	cellular senescence	5/207	93/1872	0.003724	0.033275	0.025769	5
GO:0071241	BP	GO:0071241	cellular response to inorganic substance	8/207	226/187	0.003736	0.03329	0.025781	8
GO:0002705	BP	GO:0002705	positive regulation of leukocyte mediated immunity	6/207	134/18723	0.00378	0.033595	0.026017	6
GO:0048638	BP	GO:0048638	regulation of developmental growth	10/207	330/18723	0.003805	0.033727	0.026119	10
GO:0010043	BP	GO:0010043	response to zinc ion	4/207	58/1872	0.003863	0.034072	0.026386	4
GO:0032481	BP	GO:0032481	positive regulation of type I interferon production	4/207	58/18723	0.003863	0.034072	0.026386	4
GO:0001738	BP	GO:0001738	morphogenesis of a polarized epithelium	5/207	94/1872	0.003899	0.034296	0.02656	5
GO:0001101	BP	GO:0001101	response to acid chemical	6/207	135/187	0.00392	0.034393	0.026636	6
GO:0019082	BP	GO:0019082	viral protein processing	3/207	29/1872	0.003938	0.034459	0.026687	3
GO:0033157	BP	GO:0033157	regulation of intracellular protein transport	8/207	229/18723	0.004044	0.035299	0.027337	8
GO:0008593	BP	GO:0008593	regulation of Notch signaling pathway	5/207	95/1872	0.004079	0.035516	0.027505	5
GO:0006979	BP	GO:0006979	response to oxidative stress	12/207	446/187	0.004142	0.035975	0.027861	12

GO:0045471	BP	GO:0045471	response to ethanol	6/207	137/187	0.004212	0.036486	0.028256	6
GO:1901655	BP	GO:1901655	cellular response to ketone	5/207	96/1872	0.004266	0.036856	0.028543	5
GO:0010800	BP	GO:0010800	positive regulation of peptidyl-threonine phosphorylation	3/207	30/18723	0.00434	0.037216	0.028822	3
GO:0051125	BP	GO:0051125	regulation of actin nucleation	3/207	30/1872	0.00434	0.037216	0.028822	3
GO:0070229	BP	GO:0070229	negative regulation of lymphocyte apoptotic process	3/207	30/18723	0.00434	0.037216	0.028822	3
GO:0018209	BP	GO:0018209	peptidyl-serine modification	10/207	338/187	0.004497	0.03847	0.029793	10
GO:0002699	BP	GO:0002699	positive regulation of immune effector process	8/207	235/18723	0.004719	0.040265	0.031183	8
GO:0001782	BP	GO:0001782	B cell homeostasis	3/207	31/1872	0.004766	0.040364	0.03126	3
GO:0008045	BP	GO:0008045	motor neuron axon guidance	3/207	31/1872	0.004766	0.040364	0.03126	3
GO:0090183	BP	GO:0090183	regulation of kidney development	3/207	31/1872	0.004766	0.040364	0.03126	3
GO:0002260	BP	GO:0002260	lymphocyte homeostasis	4/207	62/1872	0.004909	0.041165	0.03188	4
GO:0032613	BP	GO:0032613	interleukin-10 production	4/207	62/1872	0.004909	0.041165	0.03188	4
GO:0032653	BP	GO:0032653	regulation of interleukin-10 production	4/207	62/18723	0.004909	0.041165	0.03188	4
GO:0045428	BP	GO:0045428	regulation of nitric oxide biosynthetic process	4/207	62/18723	0.004909	0.041165	0.03188	4
GO:0007346	BP	GO:0007346	regulation of mitotic cell cycle	12/207	457/187	0.005015	0.041951	0.032488	12
GO:0061640	BP	GO:0061640	cytoskeleton-dependent cytokinesis	5/207	100/187	0.005071	0.042262	0.032729	5
GO:0001865	BP	GO:0001865	NK T cell differentiation	2/207	10/1872	0.005164	0.042262	0.032729	2
GO:0001915	BP	GO:0001915	negative regulation of T cell mediated cytotoxicity	2/207	10/18723	0.005164	0.042262	0.032729	2
GO:0002291	BP	GO:0002291	T cell activation via T cell receptor contact with antigen bound to MHC molecule on antigen presenting cell	2/207	10/18723	0.005164	0.042262	0.032729	2
GO:0019062	BP	GO:0019062	virion attachment to host cell	2/207	10/1872	0.005164	0.042262	0.032729	2

GO:0030953	BP	GO:0030953	astral microtubule organization	2/207	10/1872	0.005164	0.042262	0.032729	2
GO:0032070	BP	GO:0032070	regulation of deoxyribonuclease activity	2/207	10/18723	0.005164	0.042262	0.032729	2
GO:0060368	BP	GO:0060368	regulation of Fc receptor mediated stimulatory signaling pathway	2/207	10/18723	0.005164	0.042262	0.032729	2
GO:1902946	BP	GO:1902946	protein localization to early endosome	2/207	10/1872	0.005164	0.042262	0.032729	2
GO:0050854	BP	GO:0050854	regulation of antigen receptor-mediated signaling pathway	4/207	63/18723	0.005197	0.042393	0.032831	4
GO:0043372	BP	GO:0043372	positive regulation of CD4-positive, alpha-beta T cell differentiation	3/207	32/18723	0.005217	0.042393	0.032831	3
GO:2000515	BP	GO:2000515	negative regulation of CD4-positive, alpha-beta T cell activation	3/207	32/18723	0.005217	0.042393	0.032831	3
GO:0150115	BP	GO:0150115	cell-substrate junction organization	5/207	101/187	0.005289	0.042875	0.033204	5
GO:0001885	BP	GO:0001885	endothelial cell development	4/207	64/1872	0.005497	0.044349	0.034346	4
GO:0080164	BP	GO:0080164	regulation of nitric oxide metabolic process	4/207	64/18723	0.005497	0.044349	0.034346	4
GO:0035710	BP	GO:0035710	CD4-positive, alpha-beta T cell activation	5/207	102/187	0.005512	0.044373	0.034364	5
GO:0016570	BP	GO:0016570	histone modification	12/207	463/187	0.005549	0.044565	0.034512	12
GO:0002335	BP	GO:0002335	mature B cell differentiation	3/207	33/1872	0.005692	0.045392	0.035154	3
GO:0034110	BP	GO:0034110	regulation of homotypic cell-cell adhesion	3/207	33/18723	0.005692	0.045392	0.035154	3
GO:0045920	BP	GO:0045920	negative regulation of exocytosis	3/207	33/1872	0.005692	0.045392	0.035154	3
GO:0002449	BP	GO:0002449	lymphocyte mediated immunity	10/207	350/187	0.005719	0.045501	0.035237	10
GO:0045639	BP	GO:0045639	positive regulation of myeloid cell differentiation	5/207	103/18723	0.005743	0.045582	0.035301	5
GO:0045453	BP	GO:0045453	bone resorption	4/207	65/1872	0.005807	0.045986	0.035613	4
GO:0050777	BP	GO:0050777	negative regulation of immune response	7/207	194/187	0.005908	0.046677	0.036148	7
GO:0033674	BP	GO:0033674	positive regulation of kinase activity	12/207	467/187	0.00593	0.046743	0.036199	12

GO:0031346	BP	GO:0031346	positive regulation of cell projection organization	10/207	353/18723	0.006062	0.047308	0.036637	10
GO:2001056	BP	GO:2001056	positive regulation of cysteine-type endopeptidase activity	6/207	148/18723	0.006113	0.047308	0.036637	6
GO:0002562	BP	GO:0002562	somatic diversification of immune receptors via germline recombination within a single locus	4/207	66/18723	0.006129	0.047308	0.036637	4
GO:0016444	BP	GO:0016444	somatic cell DNA recombination	4/207	66/1872	0.006129	0.047308	0.036637	4
GO:0050766	BP	GO:0050766	positive regulation of phagocytosis	4/207	66/1872	0.006129	0.047308	0.036637	4
GO:0051893	BP	GO:0051893	regulation of focal adhesion assembly	4/207	66/1872	0.006129	0.047308	0.036637	4
GO:0090109	BP	GO:0090109	regulation of cell-substrate junction assembly	4/207	66/18723	0.006129	0.047308	0.036637	4
GO:0044319	BP	GO:0044319	wound healing, spreading of cells	3/207	34/1872	0.006193	0.047308	0.036637	3
GO:0090505	BP	GO:0090505	epiboly involved in wound healing	3/207	34/1872	0.006193	0.047308	0.036637	3
GO:2001022	BP	GO:2001022	positive regulation of response to DNA damage stimulus	5/207	105/18723	0.006224	0.047308	0.036637	5
GO:0031032	BP	GO:0031032	actomyosin structure organization	7/207	196/187	0.00624	0.047308	0.036637	7
GO:0035090	BP	GO:0035090	maintenance of apical/basal cell polarity	2/207	11/18723	0.006265	0.047308	0.036637	2
GO:0035457	BP	GO:0035457	cellular response to interferon-alpha	2/207	11/1872	0.006265	0.047308	0.036637	2
GO:0045199	BP	GO:0045199	maintenance of epithelial cell apical/basal polarity	2/207	11/18723	0.006265	0.047308	0.036637	2
GO:0045654	BP	GO:0045654	positive regulation of megakaryocyte differentiation	2/207	11/18723	0.006265	0.047308	0.036637	2
GO:0046501	BP	GO:0046501	protoporphyrinogen IX metabolic process	2/207	11/1872	0.006265	0.047308	0.036637	2
GO:0048096	BP	GO:0048096	chromatin-mediated maintenance of transcription	2/207	11/18723	0.006265	0.047308	0.036637	2
GO:0060746	BP	GO:0060746	parental behavior	2/207	11/1872	0.006265	0.047308	0.036637	2
GO:1905668	BP	GO:1905668	positive regulation of protein localization to endosome	2/207	11/18723	0.006265	0.047308	0.036637	2

GO:0002460	BP	GO:0002460	adaptive immune response based on somatic recombination of immune receptors built from immunoglobulin superfamily domains	10/207	356/18723	0.00642	0.048372	0.037461	10
GO:0070527	BP	GO:0070527	platelet aggregation	4/207	67/1872	0.006463	0.04846	0.037529	4
GO:0042116	BP	GO:0042116	macrophage activation	5/207	106/187	0.006475	0.04846	0.037529	5
GO:0071887	BP	GO:0071887	leukocyte apoptotic process	5/207	106/187	0.006475	0.04846	0.037529	5
GO:0001818	BP	GO:0001818	negative regulation of cytokine production	10/207	357/187	0.006543	0.048868	0.037845	10
GO:0050864	BP	GO:0050864	regulation of B cell activation	7/207	198/187	0.006585	0.049071	0.038002	7
GO:0031667	BP	GO:0031667	response to nutrient levels	12/207	474/187	0.006646	0.04942	0.038273	12
GO:0046640	BP	GO:0046640	regulation of alpha-beta T cell proliferation	3/207	35/18723	0.006719	0.049465	0.038307	3
GO:0048821	BP	GO:0048821	erythrocyte development	3/207	35/1872	0.006719	0.049465	0.038307	3
GO:0071108	BP	GO:0071108	protein K48-linked deubiquitination	3/207	35/1872	0.006719	0.049465	0.038307	3
GO:0090504	BP	GO:0090504	epiboly	3/207	35/1872	0.006719	0.049465	0.038307	3
GO:2001236	BP	GO:2001236	regulation of extrinsic apoptotic signaling pathway	6/207	151/18723	0.006725	0.049465	0.038307	6
GO:0051052	BP	GO:0051052	regulation of DNA metabolic process	10/207	359/187	0.006795	0.049875	0.038625	10
GO:0005747	CC	GO:0005747	mitochondrial respiratory chain complex I	13/216	48/19550	3.49E-15	3.76E-13	2.89E-13	13
GO:0030964	CC	GO:0030964	NADH dehydrogenase complex	13/216	48/19550	3.49E-15	3.76E-13	2.89E-13	13
GO:0045271	CC	GO:0045271	respiratory chain complex I	13/216	48/1955	3.49E-15	3.76E-13	2.89E-13	13
GO:0070469	CC	GO:0070469	respirasome	15/216	100/195	3.03E-13	2.45E-11	1.88E-11	15
GO:0005746	CC	GO:0005746	mitochondrial respirasome	14/216	89/1955	9.73E-13	6.14E-11	4.73E-11	14
GO:0098803	CC	GO:0098803	respiratory chain complex	14/216	90/1955	1.14E-12	6.14E-11	4.73E-11	14
GO:1990204	CC	GO:1990204	oxidoreductase complex	13/216	112/195	3.31E-10	1.53E-08	1.17E-08	13

GO:0098800	CC	GO:0098800	inner mitochondrial membrane protein complex	14/216	147/19550	9.68E-10	3.91E-08	3.01E-08	14
GO:0005925	CC	GO:0005925	focal adhesion	21/216	418/195	8.56E-09	3.07E-07	2.36E-07	21
GO:0030055	CC	GO:0030055	cell-substrate junction	21/216	425/195	1.14E-08	3.69E-07	2.84E-07	21
GO:0005743	CC	GO:0005743	mitochondrial inner membrane	22/216	495/195	3.37E-08	9.89E-07	7.61E-07	22
GO:0031209	CC	GO:0031209	SCAR complex	5/216	12/1955	1.17E-07	3.15E-06	2.42E-06	5
GO:0030027	CC	GO:0030027	lamellipodium	13/216	202/195	4.11E-07	1.02E-05	7.85E-06	13
GO:0101002	CC	GO:0101002	ficolin-1-rich granule	12/216	185/195	1.06E-06	2.45E-05	1.89E-05	12
GO:0098798	CC	GO:0098798	mitochondrial protein-containing complex	14/216	268/19550	1.80E-06	3.88E-05	2.98E-05	14
GO:0030667	CC	GO:0030667	secretory granule membrane	14/216	311/195	1.00E-05	0.000202	0.000155	14
GO:0070820	CC	GO:0070820	tertiary granule	10/216	164/195	1.47E-05	0.000279	0.000215	10
GO:0031252	CC	GO:0031252	cell leading edge	16/216	422/195	1.98E-05	0.000356	0.000274	16
GO:0030175	CC	GO:0030175	filopodium	8/216	107/195	2.49E-05	0.000423	0.000326	8
GO:0016514	CC	GO:0016514	SWI/SNF complex	4/216	19/1955	4.93E-05	0.000796	0.000613	4
GO:1902495	CC	GO:1902495	transmembrane transporter complex	14/216	366/195	6.01E-05	0.000924	0.000711	14
GO:0005902	CC	GO:0005902	microvillus	7/216	91/1955	6.73E-05	0.000987	0.000759	7
GO:0098562	CC	GO:0098562	cytoplasmic side of membrane	10/216	197/195	7.04E-05	0.000988	0.00076	10
GO:1990351	CC	GO:1990351	transporter complex	14/216	381/195	9.21E-05	0.001187	0.000913	14
GO:0045121	CC	GO:0045121	membrane raft	13/216	335/195	9.55E-05	0.001187	0.000913	13
GO:0098857	CC	GO:0098857	membrane microdomain	13/216	335/195	9.55E-05	0.001187	0.000913	13
GO:0009898	CC	GO:0009898	cytoplasmic side of plasma membrane	9/216	172/195	0.000129	0.001541	0.001185	9
GO:0035861	CC	GO:0035861	site of double-strand break	6/216	73/1955	0.000156	0.001737	0.001336	6
GO:0070821	CC	GO:0070821	tertiary granule membrane	6/216	73/1955	0.000156	0.001737	0.001336	6
GO:0098858	CC	GO:0098858	actin-based cell projection	10/216	221/195	0.000181	0.001951	0.001501	10
GO:0031528	CC	GO:0031528	microvillus membrane	4/216	27/1955	0.000208	0.002171	0.00167	4

GO:0071564	CC	GO:0071564	npBAF complex	3/216	12/1955	0.000272	0.002712	0.002086	3
GO:0002102	CC	GO:0002102	podosome	4/216	29/1955	0.000277	0.002712	0.002086	4
GO:1990907	CC	GO:1990907	beta-catenin-TCF complex	3/216	13/1955	0.000351	0.00333	0.002561	3
GO:0042581	CC	GO:0042581	specific granule	8/216	160/195	0.000414	0.003819	0.002937	8
GO:1904813	CC	GO:1904813	ficolin-1-rich granule lumen	7/216	124/195	0.00046	0.004131	0.003177	7
GO:0035579	CC	GO:0035579	specific granule membrane	6/216	91/1955	0.000518	0.004522	0.003478	6
GO:0071565	CC	GO:0071565	nBAF complex	3/216	15/1955	0.000549	0.004664	0.003587	3
GO:0101003	CC	GO:0101003	ficolin-1-rich granule membrane	5/216	61/1955	0.000566	0.004685	0.003603	5
GO:0090734	CC	GO:0090734	site of DNA damage	6/216	97/1955	0.000727	0.005871	0.004515	6
GO:0032433	CC	GO:0032433	filopodium tip	3/216	17/1955	0.000807	0.006355	0.004888	3
GO:0034774	CC	GO:0034774	secretory granule lumen	11/216	322/195	0.000948	0.00729	0.005607	11
GO:0060205	CC	GO:0060205	cytoplasmic vesicle lumen	11/216	325/195	0.001022	0.007675	0.005903	11
GO:0031983	CC	GO:0031983	vesicle lumen	11/216	327/195	0.001074	0.007881	0.006061	11
GO:0001772	CC	GO:0001772	immunological synapse	4/216	44/1955	0.001392	0.00999	0.007683	4
GO:0005884	CC	GO:0005884	actin filament	6/216	114/195	0.001681	0.011804	0.009078	6
GO:0097197	CC	GO:0097197	tetraspanin-enriched microdomain	2/216	10/1955	0.005158	0.035446	0.027262	2
GO:0031234	CC	GO:0031234	extrinsic component of cytoplasmic side of plasma membrane	5/216	101/19550	0.005281	0.035535	0.02733	5
GO:0002178	CC	GO:0002178	palmitoyltransferase complex	2/216	11/1955	0.006258	0.041253	0.031728	2
GO:0005759	CC	GO:0005759	mitochondrial matrix	12/216	480/195	0.00731	0.045437	0.034946	12
GO:0005938	CC	GO:0005938	cell cortex	9/216	308/195	0.007448	0.045437	0.034946	9
GO:0018995	CC	GO:0018995	host cellular component	2/216	12/1955	0.007456	0.045437	0.034946	2
GO:0043657	CC	GO:0043657	host cell	2/216	12/1955	0.007456	0.045437	0.034946	2
GO:0070603	CC	GO:0070603	SWI/SNF superfamily-type complex	4/216	72/19550	0.008301	0.049568	0.038123	4
GO:1904949	CC	GO:1904949	ATPase complex	4/216	73/1955	0.008708	0.049568	0.038123	4

GO:0001931	CC	GO:0001931	uropod	2/216	13/1955	0.008747	0.049568	0.038123	2
GO:0031254	CC	GO:0031254	cell trailing edge	2/216	13/1955	0.008747	0.049568	0.038123	2
GO:0003954	MF	GO:0003954	NADH dehydrogenase activity	13/212	45/18368	2.35E-15	1.02E-12	8.41E-13	13
GO:0008137	MF	GO:0008137	NADH dehydrogenase (ubiquinone) activity	12/212	42/18368	3.34E-14	6.59E-12	5.46E-12	12
GO:0050136	MF	GO:0050136	NADH dehydrogenase (quinone) activity	12/212	43/18368	4.58E-14	6.59E-12	5.46E-12	12
GO:0003955	MF	GO:0003955	NAD(P)H dehydrogenase (quinone) activity	12/212	45/18368	8.42E-14	9.09E-12	7.53E-12	12
GO:0015453	MF	GO:0015453	oxidoreduction-driven active transmembrane transporter activity	13/212	72/18368	1.74E-12	1.32E-10	1.09E-10	13
GO:0016655	MF	GO:0016655	oxidoreductase activity, acting on NAD(P)H, quinone or similar compound as acceptor	12/212	57/18368	1.83E-12	1.32E-10	1.09E-10	12
GO:0016651	MF	GO:0016651	oxidoreductase activity, acting on NAD(P)H	13/212	88/18368	2.50E-11	1.54E-09	1.28E-09	13
GO:0009055	MF	GO:0009055	electron transfer activity	14/212	126/183	2.14E-10	1.16E-08	9.57E-09	14
GO:0003712	MF	GO:0003712	transcription coregulator activity	24/212	489/183	2.53E-09	1.21E-07	1.00E-07	24
GO:0015399	MF	GO:0015399	primary active transmembrane transporter activity	13/212	181/18368	1.88E-07	8.13E-06	6.73E-06	13
GO:0003713	MF	GO:0003713	transcription coactivator activity	15/212	262/183	4.09E-07	1.60E-05	1.33E-05	15
GO:0051015	MF	GO:0051015	actin filament binding	11/212	217/183	4.57E-05	0.001645	0.001363	11
GO:0022804	MF	GO:0022804	active transmembrane transporter activity	15/212	406/183	7.86E-05	0.002612	0.002164	15
GO:0051539	MF	GO:0051539	4 iron, 4 sulfur cluster binding	5/212	42/1836	0.000117	0.003376	0.002797	5
GO:0140103	MF	GO:0140103	catalytic activity, acting on a glycoprotein	4/212	23/18368	0.000129	0.003376	0.002797	4
GO:0051536	MF	GO:0051536	iron-sulfur cluster binding	6/212	68/1836	0.000133	0.003376	0.002797	6
GO:0051540	MF	GO:0051540	metal cluster binding	6/212	68/1836	0.000133	0.003376	0.002797	6
GO:0001784	MF	GO:0001784	phosphotyrosine residue binding	5/212	44/1836	0.000147	0.003531	0.002925	5

GO:0005200	MF	GO:0005200	structural constituent of cytoskeleton	7/212	103/183	0.000192	0.004195	0.003475	7
GO:0003779	MF	GO:0003779	actin binding	15/212	441/183	0.000195	0.004195	0.003475	15
GO:0140297	MF	GO:0140297	DNA-binding transcription factor binding	14/212	394/18368	0.000204	0.004195	0.003475	14
GO:0045309	MF	GO:0045309	protein phosphorylated amino acid binding	5/212	56/18368	0.000463	0.00876	0.007258	5
GO:0019902	MF	GO:0019902	phosphatase binding	9/212	196/183	0.000466	0.00876	0.007258	9
GO:0051219	MF	GO:0051219	phosphoprotein binding	6/212	88/1836	0.000543	0.009782	0.008104	6
GO:0042826	MF	GO:0042826	histone deacetylase binding	7/212	127/183	0.000686	0.011849	0.009816	7
GO:0030295	MF	GO:0030295	protein kinase activator activity	6/212	101/183	0.001123	0.01866	0.015459	6
GO:0031490	MF	GO:0031490	chromatin DNA binding	6/212	103/183	0.001243	0.019891	0.016479	6
GO:0019209	MF	GO:0019209	kinase activator activity	6/212	107/183	0.001513	0.02334	0.019336	6
GO:0008373	MF	GO:0008373	sialyltransferase activity	3/212	21/1836	0.001729	0.025554	0.021171	3
GO:0004715	MF	GO:0004715	non-membrane spanning protein tyrosine kinase activity	4/212	45/18368	0.001775	0.025554	0.021171	4
GO:0051020	MF	GO:0051020	GTPase binding	10/212	291/183	0.002104	0.029319	0.024289	10
GO:0016746	MF	GO:0016746	acyltransferase activity	9/212	250/183	0.002551	0.033014	0.027351	9
GO:0043274	MF	GO:0043274	phospholipase binding	3/212	24/1836	0.002566	0.033014	0.027351	3
GO:0019887	MF	GO:0019887	protein kinase regulator activity	8/212	204/183	0.002598	0.033014	0.027351	8
GO:0031267	MF	GO:0031267	small GTPase binding	9/212	259/183	0.003227	0.039836	0.033003	9
GO:0017124	MF	GO:0017124	SH3 domain binding	6/212	130/183	0.004015	0.048179	0.039915	6
