# Supplementary material for: Prognostic Signature Development on the Basis of Macrophage Phagocytosis-Mediated Oxidative Phosphorylation in Bladder Cancer
Source: Oxid Med Cell Longev. 2022 Sep 29;2022:4754935. doi: 10.1155/2022/4754935 (PMC9537622; doi:10.1155/2022/4754935)
Supplement: Supplementary 9 — Supplementary Table 5: the list of enriched KEGG pathways of the MPOP. [file 4754935.f9.pdf]

	ONTO	ID	Description	GeneRa	BgRatio	pvalue	p.adjust	qvalue	Count
hsa04714	KEGG	hsa04714	Thermogenesis	23/138	232/814	4.90E-12	1.10E-09	7.94E-10	23
hsa05130	KEGG	hsa05130	Pathogenic Escherichia coli infection	19/138	197/814	6.71E-10	5.24E-08	3.77E-08	19
hsa0466	KE	hsa0466	Fc gamma R-mediated	14/1	97/8	6.98E-10	5.24E-08	3.77E-08	14
hsa05208	KEGG	hsa05208	Chemical carcinogenesis - reactive oxygen species	19/138	223/814	5.44E-09	3.06E-07	2.21E-07	19
hsa05415	KEGG	hsa05415	Diabetic cardiomyopathy	18/138	203/814	7.57E-09	3.41E-07	2.46E-07	18
hsa04810	KEGG	hsa04810	Regulation of actin cytoskeleton	18/138	218/814	2.33E-08	7.87E-07	5.67E-07	18
hsa04723	KEGG	hsa04723	Retrograde endocannabinoid signaling	15/138	148/814	2.45E-08	7.87E-07	5.67E-07	15
hsa04932	KEGG	hsa04932	Non-alcoholic fatty liver disease	15/138	155/814	4.58E-08	1.25E-06	9.02E-07	15
hsa00190	KEGG	hsa00190	Oxidative phosphorylation	14/138	134/814	5.01E-08	1.25E-06	9.02E-07	14
hsa05020	KEGG	hsa05020	Prion disease	19/138	273/814	1.44E-07	3.25E-06	2.34E-06	19
hsa05012	KEGG	hsa05012	Parkinson disease	18/138	266/814	4.81E-07	9.85E-06	7.10E-06	18
hsa05010	KEGG	hsa05010	Alzheimer disease	21/138	384/814	1.70E-06	3.18E-05	2.29E-05	21
hsa05014	KEGG	hsa05014	Amyotrophic lateral sclerosis	20/138	364/814	2.87E-06	4.83E-05	3.48E-05	20
hsa05135	KEGG	hsa05135	Yersinia infection	12/138	137/814	3.16E-06	4.83E-05	3.48E-05	12
hsa04670	KEGG	hsa04670	Leukocyte transendothelial migration	11/138	114/814	3.22E-06	4.83E-05	3.48E-05	11
hsa05132	KEGG	hsa05132	Salmonella infection	16/138	249/814	4.30E-06	6.05E-05	4.36E-05	16
hsa04650	KEGG	hsa04650	Natural killer cell mediated cytotoxicity	11/138	131/814	1.24E-05	0.000165	0.000119	11
hsa05016	KEGG	hsa05016	Huntington disease	17/138	306/814	1.48E-05	0.000185	0.000133	17
hsa05131	KEGG	hsa05131	Shigellosis	15/138	247/814	1.72E-05	0.000204	0.000147	15
hsa0051	KE	hsa0051	Mucin type O-glycan	6/13	36/8	2.72E-05	0.000306	0.000221	6
hsa0510	KE	hsa0510	Bacterial invasion of	8/13	77/8	4.36E-05	0.000467	0.000337	8
hsa05022	KEGG	hsa05022	Pathways of neurodegeneration -	21/138	476/814	4.61E-05	0.000472	0.00034	21
hsa0466	KE	hsa0466	B cell receptor signaling	8/13	82/8	6.88E-05	0.000673	0.000485	8
hsa05166	KEGG	hsa05166	Human T-cell leukemia virus 1 infection	13/138	222/814	9.38E-05	0.00088	0.000634	13
hsa05235	KEGG	hsa05235	PD-L1 expression and PD-1 checkpoint pathway in	8/138	89/8144	0.000123	0.001111	0.000801	8
hsa04380	KEGG	hsa04380	Osteoclast differentiation	9/138	128/814	0.000307	0.002657	0.001914	9
hsa0541	KE	hsa0541	Viral myocarditis	6/13	60/8	0.000501	0.004178	0.00301	6

hsa0453 0	KE GG	hsa0453 0	Tight junction	10/1 38	169/ 814	0.00057	0.004583	0.003302	10
hsa0465	KE	hsa0465	Th1 and Th2 cell	7/13	92/8	0.00091	0.007062	0.005088	7
hsa0452	KE	hsa0452	Adherens junction	6/13	71/8	0.001232	0.00924	0.006657	6
hsa0461 3	KE GG	hsa0461 3	Neutrophil extracellular trap formation	10/1 38	190/ 814	0.001404	0.010193	0.007343	10
hsa0414 0	KE GG	hsa0414 0	Autophagy - animal	8/13 8	141/ 814	0.002651	0.018638	0.013428	8
hsa0415 0	KE GG	hsa0415 0	mTOR signaling pathway	8/13 8	156/ 814	0.004914	0.033506	0.02414	8
hsa0406 8	KE GG	hsa0406 8	FoxO signaling pathway	7/13 8	131/ 814	0.006728	0.044243	0.031876	7
hsa0493 3	KE GG	hsa0493 3	AGE-RAGE signaling pathway in diabetic	6/13 8	100/ 814	0.006882	0.044243	0.031876	6
hsa0516 9	KE GG	hsa0516 9	Epstein-Barr virus infection	9/13 8	202/ 814	0.007243	0.045272	0.032617	9
hsa0520 5	KE GG	hsa0520 5	Proteoglycans in cancer	9/13 8	205/ 814	0.007949	0.048337	0.034825	9
hsa0514	KE	hsa0514	Leishmaniasis	5/13	77/8	0.009679	0.056756	0.040891	5
hsa0514	KE	hsa0514	Malaria	4/13	50/8	0.009949	0.056756	0.040891	4
hsa0516 5	KE GG	hsa0516 5	Human papillomavirus infection	12/1 38	331/ 814	0.010271	0.056756	0.040891	12
hsa0006	KE	hsa0006	Fatty acid elongation	3/13	27/8	0.010342	0.056756	0.040891	3
hsa0515 2	KE GG	hsa0515 2	Tuberculosis	8/13 8	180/ 814	0.011299	0.06053	0.04361	8
hsa0451 4	KE GG	hsa0451 4	Cell adhesion molecules	7/13 8	149/ 814	0.013169	0.06891	0.049647	7
hsa0516 3	KE GG	hsa0516 3	Human cytomegalovirus infection	9/13 8	225/ 814	0.014074	0.071967	0.05185	9
hsa0407 1	KE GG	hsa0407 1	Sphingolipid signaling pathway	6/13 8	119/ 814	0.015456	0.075602	0.054469	6
hsa0472 2	KE GG	hsa0472 2	Neurotrophin signaling pathway	6/13 8	119/ 814	0.015456	0.075602	0.054469	6
hsa0421 8	KE GG	hsa0421 8	Cellular senescence	7/13 8	156/ 814	0.016593	0.075734	0.054564	7
hsa0520 2	KE GG	hsa0520 2	Transcriptional misregulation in cancer	8/13 8	193/ 814	0.016634	0.075734	0.054564	8
hsa0491 9	KE GG	hsa0491 9	Thyroid hormone signaling pathway	6/13 8	121/ 814	0.016659	0.075734	0.054564	6
hsa0516 7	KE GG	hsa0516 7	Kaposi sarcoma-associated herpesvirus infection	8/13 8	194/ 814	0.017109	0.075734	0.054564	8
hsa0433	KE	hsa0433	Notch signaling pathway	4/13	59/8	0.017503	0.075734	0.054564	4
hsa0437	KE	hsa0437	VEGF signaling pathway	4/13	59/8	0.017503	0.075734	0.054564	4
hsa0053 3	KE GG	hsa0053 3	Glycosaminoglycan biosynthesis - keratan	2/13 8	14/8 144	0.022703	0.096173	0.06929	2
hsa0515	KE	hsa0515	Staphylococcus aureus	5/13	96/8	0.023246	0.096173	0.06929	5
hsa0522 5	KE GG	hsa0522 5	Hepatocellular carcinoma	7/13 8	168/ 814	0.023857	0.096173	0.06929	7
hsa0125	KE	hsa0125	Biosynthesis of nucleotide	3/13	37/8	0.024309	0.096173	0.06929	3
hsa0523	KE	hsa0523	Choline metabolism in	5/13	98/8	0.025151	0.096173	0.06929	5
hsa0060 3	KE GG	hsa0060 3	Glycosphingolipid biosynthesis - globo and	2/13 8	15/8 144	0.025909	0.096173	0.06929	2

hsa0060	KE	hsa0060	Glycosphingolipid	2/13	15/8	0.025909	0.096173	0.06929	2
hsa0516 4	KE GG	hsa0516 4	Influenza A	7/13 8	171/ 814	0.025969	0.096173	0.06929	7
hsa0534	KE	hsa0534	Primary immunodeficiency	3/13	38/8	0.026074	0.096173	0.06929	3
hsa0466	KE	hsa0466	Fc epsilon RI signaling	4/13	68/8	0.027897	0.10124	0.07294	4
hsa0462 5	KE GG	hsa0462 5	C-type lectin receptor signaling pathway	5/13 8	104/ 814	0.031457	0.110593	0.079679	5
hsa0466 0	KE GG	hsa0466 0	T cell receptor signaling pathway	5/13 8	104/ 814	0.031457	0.110593	0.079679	5
hsa0436 0	KE GG	hsa0436 0	Axon guidance	7/13 8	182/ 814	0.034805	0.119226	0.085899	7
hsa0411	KE	hsa0411	p53 signaling pathway	4/13	73/8	0.034973	0.119226	0.085899	4
hsa0513	KE	hsa0513	Pertussis	4/13	76/8	0.039675	0.131276	0.09458	4
hsa0521	KE	hsa0521	Pancreatic cancer	4/13	76/8	0.039675	0.131276	0.09458	4
hsa0466 8	KE GG	hsa0466 8	TNF signaling pathway	5/13 8	112/ 814	0.041291	0.134643	0.097006	5
hsa0152	KE	hsa0152	EGFR tyrosine kinase	4/13	79/8	0.04472	0.143743	0.103562	4
hsa0052	KE	hsa0052	Amino sugar and	3/13	49/8	0.04984	0.157943	0.113793	3
hsa0521	KE	hsa0521	Colorectal cancer	4/13	86/8	0.057826	0.180706	0.130193	4
hsa0461 1	KE GG	hsa0461 1	Platelet activation	5/13 8	124/ 814	0.05919	0.182434	0.131438	5
hsa0401 5	KE GG	hsa0401 5	Rap1 signaling pathway	7/13 8	210/ 814	0.065665	0.198265	0.142844	7
hsa0078	KE	hsa0078	Lipoic acid metabolism	1/13	4/81	0.066088	0.198265	0.142844	1
hsa0517 0	KE GG	hsa0517 0	Human immunodeficiency virus 1 infection	7/13 8	212/ 814	0.068353	0.20236	0.145794	7
hsa0121	KE	hsa0121	Fatty acid metabolism	3/13	57/8	0.071901	0.209912	0.151235	3
hsa0532	KE	hsa0532	Rheumatoid arthritis	4/13	93/8	0.07277	0.209912	0.151235	4
hsa0521	KE	hsa0521	Endometrial cancer	3/13	58/8	0.074921	0.213384	0.153736	3
hsa0104	KE	hsa0104	Biosynthesis of unsaturated	2/13	27/8	0.075976	0.213681	0.153951	2
hsa0521	KE	hsa0521	Prostate cancer	4/13	97/8	0.08211	0.228084	0.164327	4
hsa0516 2	KE GG	hsa0516 2	Measles	5/13 8	139/ 814	0.086906	0.235589	0.169735	5
hsa0541 8	KE GG	hsa0541 8	Fluid shear stress and atherosclerosis	5/13 8	139/ 814	0.086906	0.235589	0.169735	5
hsa0491 4	KE GG	hsa0491 4	Progesterone-mediated oocyte maturation	4/13 8	102/ 814	0.094575	0.253326	0.182513	4
hsa0406 4	KE GG	hsa0406 4	NF-kappa B signaling pathway	4/13 8	104/ 814	0.099799	0.264173	0.190328	4
hsa0522	KE	hsa0522	Acute myeloid leukemia	3/13	67/8	0.1045	0.273401	0.196977	3
hsa0406 2	KE GG	hsa0406 2	Chemokine signaling pathway	6/13 8	192/ 814	0.107362	0.277661	0.200046	6
hsa0465 9	KE GG	hsa0465 9	Th17 cell differentiation	4/13 8	108/ 814	0.110639	0.279043	0.201041	4
hsa0493 1	KE GG	hsa0493 1	Insulin resistance	4/13 8	108/ 814	0.110639	0.279043	0.201041	4
hsa0521	KE	hsa0521	Renal cell carcinoma	3/13	69/8	0.111617	0.279043	0.201041	3
hsa0512 0	KE GG	hsa0512 0	Epithelial cell signaling in Helicobacter pylori	3/13 8	70/8 144	0.115244	0.281847	0.203062	3
hsa0523	KE	hsa0523	Central carbon metabolism	3/13	70/8	0.115244	0.281847	0.203062	3
hsa0514 5	KE GG	hsa0514 5	Toxoplasmosis	4/13 8	112/ 814	0.121983	0.29512	0.212624	4

hsa05160	KEGG	hsa05160	Hepatitis C	5/138	157/814	0.127636	0.303454	0.218629	5
hsa0514	KE	hsa0514	African trypanosomiasis	2/13	37/8	0.129474	0.303454	0.218629	2
hsa0521	KE	hsa0521	Thyroid cancer	2/13	37/8	0.129474	0.303454	0.218629	2
hsa05203	KEGG	hsa05203	Viral carcinogenesis	6/138	204/814	0.132429	0.303836	0.218904	6
hsa04217	KEGG	hsa04217	Necroptosis	5/138	159/814	0.132629	0.303836	0.218904	5
hsa04144	KEGG	hsa04144	Endocytosis	7/138	251/814	0.133688	0.303836	0.218904	7
hsa0497	KE	hsa0497	Gastric acid secretion	3/13	76/8	0.1379	0.307203	0.22133	3
hsa0522	KE	hsa0522	Chronic myeloid leukemia	3/13	76/8	0.1379	0.307203	0.22133	3
hsa05161	KEGG	hsa05161	Hepatitis B	5/138	162/814	0.140281	0.309443	0.222944	5
hsa04151	KEGG	hsa04151	PI3K-Akt signaling pathway	9/138	354/814	0.146168	0.319298	0.230044	9
hsa0521	KE	hsa0521	Bladder cancer	2/13	41/8	0.152775	0.328832	0.236913	2
hsa04310	KEGG	hsa04310	Wnt signaling pathway	5/138	167/814	0.153455	0.328832	0.236913	5
hsa05417	KEGG	hsa05417	Lipid and atherosclerosis	6/138	215/814	0.157648	0.334629	0.24109	6
hsa0086	KE	hsa0086	Porphyrin metabolism	2/13	43/8	0.164716	0.346365	0.249544	2
hsa04114	KEGG	hsa04114	Oocyte meiosis	4/138	131/814	0.181928	0.37758	0.272034	4
hsa0493	KE	hsa0493	Type II diabetes mellitus	2/13	46/8	0.182916	0.37758	0.272034	2
hsa0051	KE	hsa0051	Other types of O-glycan	2/13	47/8	0.189049	0.386691	0.278598	2
hsa04210	KEGG	hsa04210	Apoptosis	4/138	136/814	0.199114	0.398949	0.28743	4
hsa04014	KEGG	hsa04014	Ras signaling pathway	6/138	232/814	0.200361	0.398949	0.28743	6
hsa05171	KEGG	hsa05171	Coronavirus disease - COVID-19	6/138	232/814	0.200361	0.398949	0.28743	6
hsa0522	KE	hsa0522	Small cell lung cancer	3/13	92/8	0.204438	0.403496	0.290706	3
hsa0051	KE	hsa0051	N-Glycan biosynthesis	2/13	50/8	0.207604	0.405052	0.291827	2
hsa0491	KE	hsa0491	GnRH signaling pathway	3/13	93/8	0.208827	0.405052	0.291827	3
hsa0465	KE	hsa0465	IL-17 signaling pathway	3/13	94/8	0.213237	0.410071	0.295443	3
hsa04120	KEGG	hsa04120	Ubiquitin mediated proteolysis	4/138	142/814	0.220357	0.416642	0.300177	4
hsa04936	KEGG	hsa04936	Alcoholic liver disease	4/138	142/814	0.220357	0.416642	0.300177	4
hsa04010	KEGG	hsa04010	MAPK signaling pathway	7/138	294/814	0.231061	0.433239	0.312134	7
hsa04072	KEGG	hsa04072	Phospholipase D signaling pathway	4/138	148/814	0.242178	0.450331	0.324449	4
hsa05142	KEGG	hsa05142	Chagas disease	3/138	102/814	0.249172	0.459539	0.331083	3
hsa04510	KEGG	hsa04510	Focal adhesion	5/138	201/814	0.254521	0.464948	0.33498	5
hsa04145	KEGG	hsa04145	Phagosome	4/138	152/814	0.256994	0.464948	0.33498	4
hsa04620	KEGG	hsa04620	Toll-like receptor signaling pathway	3/138	104/814	0.258305	0.464948	0.33498	3

hsa04921	KEGG	hsa04921	Oxytocin signaling pathway	4/138	154/814	0.264472	0.467838	0.337062	4
hsa0006	KE	hsa0006	Fatty acid biosynthesis	1/13	18/8	0.265046	0.467838	0.337062	1
hsa04928	KEGG	hsa04928	Parathyroid hormone synthesis, secretion and	3/138	106/814	0.267482	0.467838	0.337062	3
hsa04934	KEGG	hsa04934	Cushing syndrome	4/138	155/814	0.268227	0.467838	0.337062	4
hsa0473	KE	hsa0473	Long-term depression	2/13	60/8	0.270426	0.468045	0.337212	2
hsa04390	KEGG	hsa04390	Hippo signaling pathway	4/138	157/814	0.275766	0.469502	0.338261	4
hsa0053	KE	hsa0053	Glycosaminoglycan	1/13	19/8	0.277528	0.469502	0.338261	1
hsa0121	KE	hsa0121	2-Oxocarboxylic acid	1/13	19/8	0.277528	0.469502	0.338261	1
hsa05207	KEGG	hsa05207	Chemical carcinogenesis - receptor activation	5/138	212/814	0.290327	0.487489	0.35122	5
hsa04630	KEGG	hsa04630	JAK-STAT signaling pathway	4/138	162/814	0.294761	0.491268	0.353943	4
