# Supplementary material for: Prognostic Signature Development on the Basis of Macrophage Phagocytosis-Mediated Oxidative Phosphorylation in Bladder Cancer
Source: Oxid Med Cell Longev. 2022 Sep 29;2022:4754935. doi: 10.1155/2022/4754935 (PMC9537622; doi:10.1155/2022/4754935)
Supplement: Supplementary 10 — Supplementary Table 6: the list of MRPs differentially expressed in bladder cancer. [file 4754935.f10.pdf]

| Gene | P value |
| --- | --- |
| IER5L | 1.3E-10 |
| NFIA | 2.6E-10 |
| GTPBP3 | 2.7E-10 |
| PPAP2B | 5.3E-10 |
| CMC1 | 2.1E-09 |
| KIF23 | 2.4E-09 |
| TIMMDC1 | 4.1E-09 |
| HMBS | 5.2E-09 |
| LAMTOR2 | 0.000000006 |
| TACO1 | 0.000000014 |
| PTDSS1 | 0.000000018 |
| CIT | 0.000000021 |
| HIC1 | 0.000000025 |
| CDK2 | 0.00000003 |
| SLC25A1 | 0.000000034 |
| RNF122 | 0.00000007 |
| MECR | 0.00000012 |
| SIX4 | 0.00000016 |
| OTUB1 | 0.00000021 |
| KLF6 | 0.00000031 |
| EMC1 | 0.00000035 |
| ARPC3 | 0.00000044 |
| DOLPP1 | 0.00000049 |
| PDCD10 | 0.00000051 |
| FADD | 0.00000058 |
| QPCTL | 0.0000006 |
| FOXO1 | 0.00000085 |
| OSTC | 0.00000086 |
| TCEB1 | 0.0000013 |
| HMGB2 | 0.0000014 |
| NDUFS8 | 0.0000016 |
| NDUFBC | 0.0000017 |
| APMAP | 0.0000025 |
| ARPC4 | 0.0000029 |
| NDUFS6 | 0.0000035 |
| ZNF746 | 0.000005 |
| NDUFB4 | 0.0000067 |
| NDUAF7 | 0.0000088 |
| NDUFA9 | 0.0000099 |
| UQCC1 | 0.000014 |
| ADAM10 | 0.000019 |
| LCMT1 | 0.000019 |
| MYC | 0.000022 |
| SUPT20H | 0.000026 |
| XPR1 | 0.000026 |
| NCKAP1L | 0.000031 |

| | |
|---|---|
| TMEM165 | 0.000031 |
| NDUFS2 | 0.000032 |
| AIFM1 | 0.000033 |
| CHMP1A | 0.000038 |
| TMEM119 | 0.000039 |
| DOCK11 | 0.000056 |
| MAP3K3 | 0.000062 |
| COX18 | 0.000079 |
| CD79B | 0.000081 |
| CMAS | 0.000086 |
| MAP3K10 | 0.000092 |
| ANAPC7 | 0.0001 |
| WASF2 | 0.0001 |
| NDUFB9 | 0.00014 |
| COX5B | 0.00015 |
| ALAD | 0.00016 |
| LIPT2 | 0.00019 |
| NXT1 | 0.0002 |
| TP73 | 0.0002 |
| STARD7 | 0.00021 |
| JAK1 | 0.00022 |
| JMJD1C | 0.00023 |
| SLC9A3R1 | 0.00024 |
| LIMK2 | 0.00027 |
| MGAT1 | 0.00035 |
| GRSF1 | 0.00042 |
| SLC39A9 | 0.0006 |
| HMGB1 | 0.00063 |
| CLIC4 | 0.00064 |
| VCAM1 | 0.00075 |
| C1GALT1 | 0.00097 |
| SPPL3 | 0.001 |
| ELOVL1 | 0.0011 |
| NHLRC2 | 0.0011 |
| PTEN | 0.0012 |
| C1GALT1C1 | 0.0014 |
| LAMTOR4 | 0.0014 |
| ACTR2 | 0.0021 |
| SLC25A1 | 0.0023 |
| OSR2 | 0.0027 |
| UBE2K | 0.0029 |
| ELOVL6 | 0.0029 |
| CADM1 | 0.003 |
| VPS37A | 0.0035 |
| RRAGA | 0.0037 |
| SAMD4B | 0.0038 |
| RAC1 | 0.0039 |

| | |
|---|---|
| WDR1 | 0.004 |
| RPL21 | 0.0046 |
| NDUFA1 | 0.0048 |
| TM2D1 | 0.005 |
| MTIF3 | 0.0051 |
| GNE | 0.0063 |
| NDUFV1 | 0.0065 |
| MAML2 | 0.0075 |
| ARID1B | 0.008 |
| GRHL1 | 0.0082 |
| FTN4IP1 | 0.0086 |
| FOXO4 | 0.0088 |
| STK4 | 0.0088 |
| CAB39 | 0.0089 |
| ZFX | 0.0094 |
| GTPBP6 | 0.0095 |
| ACTB | 0.0098 |
| PTPRC | 0.0099 |
| NDUFC1 | 0.012 |
| C5AR1 | 0.015 |
| UBE2J1 | 0.015 |
| UBR4 | 0.015 |
| SLC39A13 | 0.017 |
| ZNF217 | 0.017 |
| PRDM1 | 0.022 |
| SMAGP | 0.023 |
| HRCT1 | 0.024 |
| NANS | 0.024 |
| MYO9B | 0.025 |
| ZBTB7A | 0.028 |
| LPIN2 | 0.03 |
| MSN | 0.03 |
| TLE3 | 0.033 |
| AIP | 0.035 |
| PDE12 | 0.036 |
| SS18 | 0.036 |
| MOB3A | 0.043 |
| ARSB | 0.046 |
| CYFIP1 | 0.049 |
| LRRC15 | 0.049 |
| UBE2D3 | 0.054 |
| NDUFB6 | 0.055 |
| NUBPL | 0.13 |
| MUC1 | 0.25 |
| PTPN6 | 0,000049 |
